# Molecular pathways of ketamine: A systematic review of immediate and sustained effects on PTSD

**DOI:** 10.1007/s00213-025-06756-4

**Published:** 2025-03-17

**Authors:** Nathan J. Wellington, Ana P. Boųcas, Jim Lagopoulos, Bonnie L. Quigley, Anna V. Kuballa

**Affiliations:** 1 National PTSD Research Centre, Thompson Institute, University of the Sunshine Coast (UniSC), Birtinya, QLD Australia; 2 School of Health, UniSC, Sippy Downs, QLD Australia; 3 Centre for Bioinnovation, UniSC, Sippy Downs, QLD Australia; 4 Thompson Brain and Mind Healthcare, Maroochydore, QLD Australia; 5 Sunshine Coast Hospital and Health Service, Sunshine Coast Health Institute, Birtinya, QLD Australia

**Keywords:** Ketamine, PTSD, BDNF, Epigenetics, Synaptic plasticity, NMDA, Memory reconsolidation, Gene expression, Pharmacokinetics, Pharmacodynamics

## Abstract

**Rationale:**

Existing studies predominantly focus on the molecular and neurobiological mechanisms underlying Ketamine’s acute treatment effects on post-traumatic stress disorder (PTSD). This emphasis has largely overlooked its sustained therapeutic effects, which hold significant potential for the development of targeted interventions.

**Objectives:**

This systematic review examines the pharmacokinetic and pharmacodynamic effects of ketamine on PTSD, differentiating between immediate and sustained molecular effects.

**Method:**

A comprehensive search across databases (Web of Science, Scopus, Global Health, PubMed) and grey literature yielded 317 articles, where 29 studies met the inclusion criteria. These studies included preclinical models and clinical trials, through neurotransmitter regulation, gene expression, synaptic plasticity, and neural pathways (PROSPERO ID: CRD42024582874).

**Results:**

We found accumulating evidence that the immediate effects of ketamine, which involve changes in GABA, glutamate, and glutamine levels, trigger the re-regulation of BDNF, enhancing synaptic plasticity via pathways such as TrkB and PSD-95. Other molecular influences also include c-Fos, GSK-3, HDAC, HCN1, and the modulation of hormones like CHR and ACTH, alongside immune responses (IL-6, IL-1β, TNF-α). Sustained effects arise from neurotransmitter remodulations and involve prolonged changes in gene expression. These include mTOR-mediated BDNF expression, alterations in GSK-3β, FkBP5, GFAP, ERK phosphorylation, and epigenetic modifications (DNMT3, MeCP2, H3K27me3, mir-132, mir-206, HDAC).

**Conclusion:**

These molecular changes promote long-term synaptic stability and re-regulation in key brain regions, contributing to prolonged therapeutic benefits. Understanding the sustained molecular and epigenetic mechanisms behind ketamine’s effects is critical for developing safe and effective personalised treatments, potentially leading to more effective recovery.

**Supplementary Information:**

The online version contains supplementary material available at 10.1007/s00213-025-06756-4.

## Introduction

Significant advancements in the treatment of post-traumatic stress disorder (PTSD) underscore ketamine’s potential in modulating specific molecular pathways linked to PTSD symptomology. Rodent models have primarily driven our understanding of the underlying neurobiological interactions of ketamine and their functional significance.

Biological similarities between rodent models and humans are vital for translating preclinical findings into clinical applications (Antontseva et al. 2020). Both species share conserved neurobiological pathways, particularly in regions like the hippocampus (HPC), amygdala (AMY), and prefrontal cortex (PFC), which are implicated in stress response and mood regulation (Munro et al. 2022). By comparing the results from rodent studies to human clinical trials, researchers can identify biomarkers and molecular targets influenced by ketamine, facilitating the development of more effective and personalised treatment strategies (Kavalali and Monteggia 2012).

The pathophysiology of PTSD involves complex disruptions in stress regulation, memory processing, and emotional control. Dysregulation of the hypothalamic-pituitary-adrenal (HPA) axis plays a central role, with PTSD patients often exhibiting hypocortisolism and hyperactive amygdala responses. These disruptions weaken feedback inhibition within the HPA axis, amplifying fear responses and impairing stress adaptation (Herman 2016; Liberzon and Abelson 2016). The elevated activity of corticotropin-releasing hormone (CRH) and altered glucocorticoid receptor sensitivity further exacerbate stress-related neural dysfunction.

Structural and functional impairments in key neural circuits, particularly the hippocampus, prefrontal cortex, and amygdala, contribute significantly to PTSD symptomatology. The hippocampus, essential for contextual memory and fear extinction, often shows reduced volume and connectivity in PTSD, correlating with deficits in memory recall and emotional regulation (Bremner 2007; Shin et al. 2006). These neural dysfunctions are further exacerbated by systemic inflammation, as evidenced by elevated levels of pro-inflammatory cytokines such as IL-6, TNF-α, and IL-1β (Borgonetti et al. 2023; de Oliveira et al. 2018; Hori and Kim 2019). These cytokines influence synaptic transmission and neural plasticity, compounding the cognitive and emotional challenges associated with PTSD.

Understanding these interconnected mechanisms is essential for elucidating ketamine’s therapeutic potential in PTSD. By targeting neural plasticity and modulating stress-related pathways, ketamine offers rapid symptom relief. Studies comparing its immediate and long-term effects in rodent and human models consistently demonstrate significant reductions in PTSD symptoms shortly after administration, reinforcing its role as a rapid-acting treatment. However, while short-term benefits are well-documented, the safety and efficacy of prolonged therapy warrant further investigation to optimise its long-term clinical use.

This systematic review focusses on the immediate pharmacokinetic and sustained pharmacodynamic influences of ketamine on PTSD, specifically characterising important molecular mechanisms. In the short-term response, there are the mechanisms that define the anaesthetic and analgesic effects of ketamine as it is active and metabolised within the body during the first 24 h. In the longer-term, there are molecular modulations that continue to present reduced symptoms of anxiety and depression alongside the reduction in traumatic memory reactivation and enhanced memory reconsolidation.

### Pharmacokinetics of ketamine

Ketamine, a compound structurally related to phencyclidine (PCP), is favoured in medical contexts due to its improved safety profile and shorter duration of action compared to PCP (Domino 2010; Mion 2017). Ketamine is commonly known as a racemic blend of two chiral enantiomers, S(+)-ketamine and R(-)-ketamine. However, it is possible to obtain pure forms of either ketamine enantiomer. S(+)-ketamine is more commonly used in clinical practice due to its significantly greater potency and rapid onset of antidepressant effects (Artin et al. 2022). In contrast, R(-)-ketamine has attracted increasing interest in research because of its prolonged antidepressant effects, making it a promising candidate for sustained relief in mood disorders (Chang et al. 2019; Elersic et al. 2024). While R-ketamine (arketamine) and S-ketamine (esketamine) have both been used successfully in the treatment of PTSD, their pharmacokinetic and pharmacodynamic profiles differ significantly due to variations in their molecular structures and subsequent interactions within biological systems (Hess et al. 2022; Jelen et al. 2021).

**Fig. 1:**
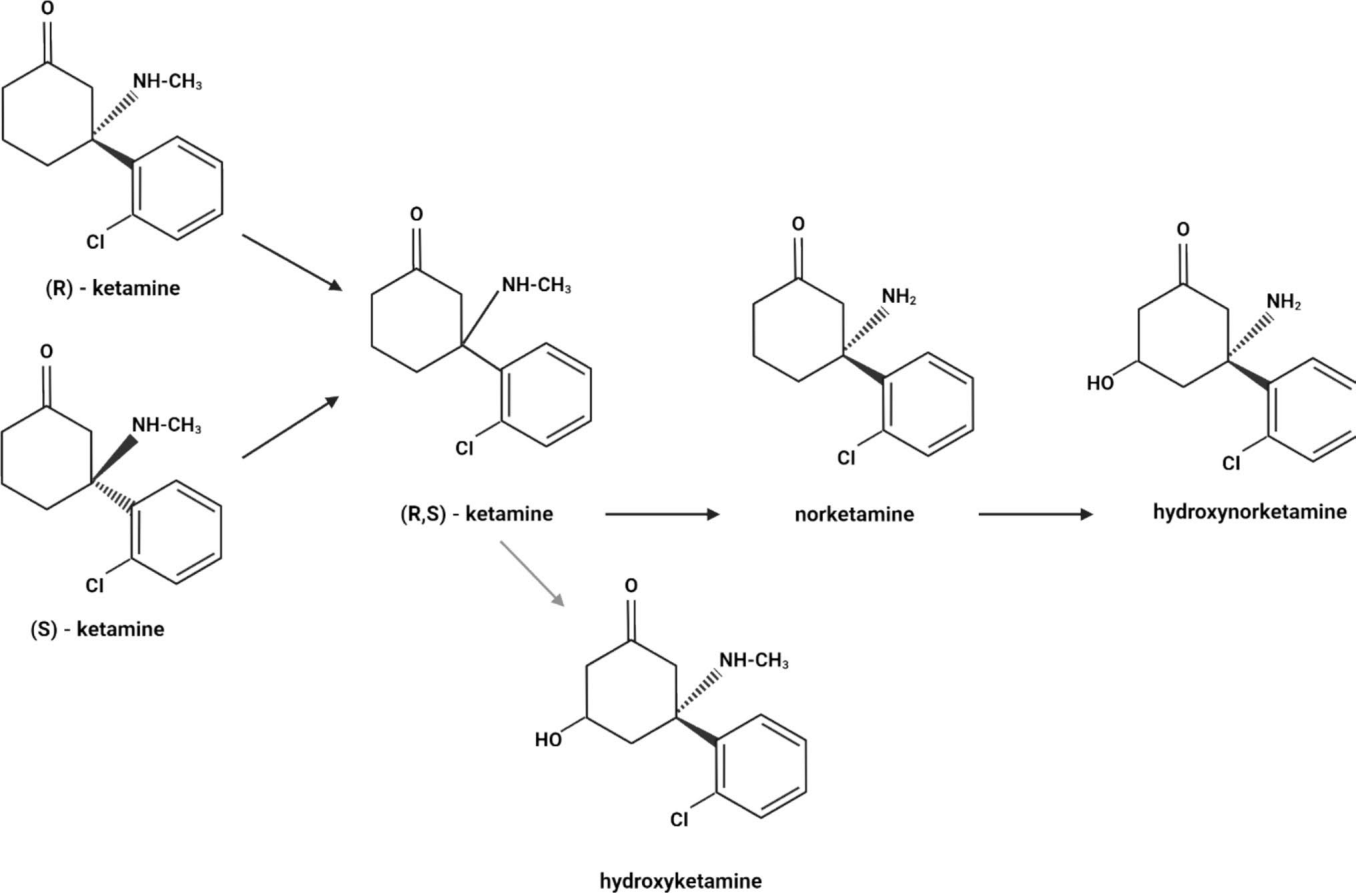
Ketamine and its metabolites. Ketamine, chemically known as 2-(2-chlorophenyl)−2-(methylamino)cyclohexanone, initially undergoes N-demethylation. This reaction, mediated by cytochrome P450 enzymes, removes the N-methyl group (NH-CH3) from the amine, resulting in norketamine, or 2-(2-chlorophenyl)−2-amino-cyclohexanone. Subsequently, norketamine undergoes hydroxylation, where a hydroxyl group (OH) is introduced at the C6 position of the cyclohexanone ring, forming hydroxynorketamine. This adds an additional functional site on the cyclohexanone ring, yielding 6-hydroxy-2-(2-chlorophenyl)−2-amino-cyclohexanone. Alternatively, ketamine can be directly hydroxylated to form hydroxyketamine. In this reaction, a hydroxyl group is added to the C6 position of the cyclohexanone ring of ketamine while retaining the N-methyl group, resulting in 6-hydroxy-2-(2-chlorophenyl)−2-(methylamino)cyclohexanone. Chemical structures built using Biorender.com and adapted from.(Dinis-Oliveira 2017; Kamp et al. 2020)

Ketamine is a lipid-soluble molecule that has a large volume of distribution, allowing it to rapidly cross the blood-brain barrier (BBB) (Dutton et al. 2023). It exhibits a low binding affinity to plasma proteins, with only 10–40% of the drug being bound to them (Gebhardt 1994). Upon oral administration, ketamine is absorbed in the gastrointestinal tract, where its lipophilic nature allows it to diffuse through lipid membranes. Both enantiomers are metabolised by cytochrome P450 enzymes (CYP3A4, CYP2B6, and CYP2C9) in the liver, which results in a relatively low bioavailability of approximately 20% (Kamp et al. 2020). Structural differences between the ketamine enantiomers result in distinct affinities for cytochrome P450 enzymes, influencing their individual rates of metabolism. Both R- and S-ketamine undergo N-demethylation to produce norketamine (NK), but S-ketamine exhibits a higher metabolic rate, resulting in a shorter duration of action compared to R-ketamine. Subsequently, NK is further hydroxylated to form hydroxynorketamine (HNK), a metabolite with its own pharmacological activity distinct from the parent compounds (Kamp et al. 2020) (Fig. 1).

The extent and rate of these metabolic transformations can vary significantly among individuals due to genetic differences in enzyme expression, impacting both the efficacy and safety of the drug. CYP2B6 polymorphisms, particularly the common CYP2B6*6 variant found in 15–60% of populations, lead to reduced enzyme activity, which affects the metabolism of drugs like ketamine and efavirenz, resulting in slower drug clearance and higher drug levels, thereby increasing the risk of adverse effects (Langmia et al. 2021). In the bloodstream, ketamine binds to plasma proteins such as albumin and α1-acid glycoprotein, which control its free concentration and facilitate its diffusion across the blood-brain barrier (GRANT 1981).

S-ketamine binds more effectively to N-methyl-D-aspartate (NMDA) receptors, resulting in stronger inhibitory effects and more noticeable dissociative experiences (Zanos and Gould 2018). This strong receptor interaction may lead to quicker changes in synaptic plasticity and neurotransmission. In contrast, R-ketamine, which binds less effectively to NMDA receptors, could promote longer-lasting neuroplastic changes and extended antidepressant benefits, possibly with fewer side effects (Liu et al. 2013).

The pharmacological implications of these differences are significant. S-ketamine’s rapid metabolism and potent NMDA receptor activity allow for effective low-dose treatments but come with an increased risk of dissociative side effects (Zanos and Gould 2018). This has led to its approval as a nasal spray for treatment-resistant depression (Rotharmel et al. 2022). In contrast, R-ketamine is under investigation for its potentially superior therapeutic profile and fewer adverse effects (Hashimoto 2020). The distinct metabolic pathways and receptor interactions of R- and S-ketamine underscore their unique roles in clinical applications, impacting their efficacy and safety profiles (Yang et al. 2015).

### Ketamine administration

**Fig. 2:**
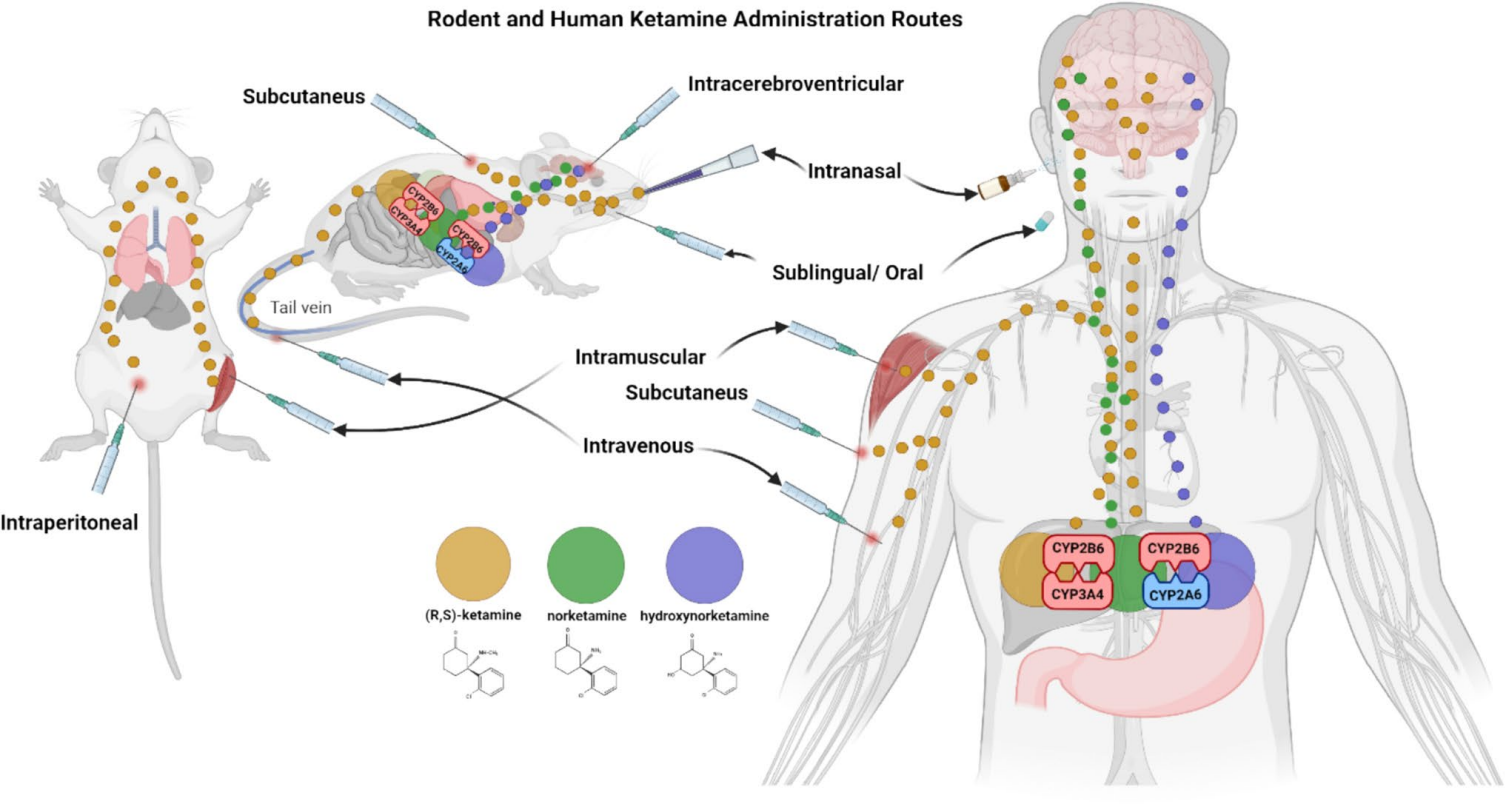
Administrative route of ketamine and the metabolic pathways (Romagnoli et al., 2017). Ketamine (shown in yellow) can be administered through various routes, such as injection or oral intake, leading to its distribution throughout the body and crossing the blood-brain barrier to exert its effects. Ketamine is primarily metabolised by cytochrome P450 enzymes (CYP2B6, CYP2A6, and CYP3A4) in the liver, where it is converted into norketamine and hydroxynorketamine. Norketamine (depicted in green) is formed through N-demethylation and further processed by the liver enzymes. Hydroxynorketamine (shown in purple) results from additional hydroxylation reactions. These metabolites, especially hydroxynorketamine, contribute to the overall pharmacological effects of ketamine, including its antidepressant properties. Figure generated us﻿ing Biorender.com

The administration of ketamine to both humans and rodents can be conducted through various methods, which affect its pharmacokinetics. These methods include sublingual/buccal/oral, subcutaneous (SC), intramuscular (IM), intravenous (IV), and intranasal (IN) routes (Berman et al. 2000; Mathisen et al. 1995). In rodent research, intraperitoneal (IP) injection is commonly used because it is simple and effective, ensuring rapid absorption into the bloodstream (Zanos et al. 2018). Rodent models also allow for brain region response investigations through intracerebroventricular (ICV) injection into the brain tissue of interest (Peltoniemi et al. 2012). Although IV administration requires more technical skill in rodents, it allows for precise dosing and immediate drug delivery, closely replicating clinical use in humans. IN administration is becoming more popular due to its non-invasive approach and potential for direct brain delivery, circumventing the blood-brain barrier (Aldrete et al. 1988) (Fig. 2).

Therapeutic administration of ketamine requires careful consideration of the risks associated with each route when deciding on the most effective and appropriate method for treatment (Table 1).

**Table 1:** Risk factors for administration routes or therapeutics (Aronson 2009; Morton et al. 2001)

Administration Route	Risk Factors
Sublingual/Buccal	- Variable absorption due to mucosal irritation. - Lower bioavailability compared to IV (due to first-pass metabolism). - Risk of mucosal discomfort or damage over time. - Potential for inconsistent dosing if saliva production is high.
Oral	- Lowest bioavailability (~ 20%) due to first-pass metabolism by the liver. - Slow onset of action. - High variability in drug absorption. - Risk of gastrointestinal side effects (nausea, vomiting).
Intranasal (IN)	- Risk of mucosal irritation or damage. - Variable absorption depending on nasal mucosa health - Risk of inconsistent dosing if nasal congestion or inflammation is present. - Potential for abuse due to ease of administration.
Subcutaneous (SC)	- Risk of local irritation or tissue necrosis at the injection site. - Slower absorption compared to IM or IV routes. - Risk of improper dosing with home administration.
Intramuscular (IM)	- Pain at the injection site. - Risk of muscle damage or infection. - Higher bioavailability than oral, but absorption can vary depending on the site of injection. - Risk of adverse psychological effects if not administered in a controlled environment.
Intravenous (IV)	- Risk of cardiovascular effects, including hypertension and tachycardia. - High potential for abuse due to rapid onset. - Requires careful monitoring to avoid overdose. - Risk of respiratory depression, especially with higher doses.

### Ketamine metabolism and elimination

The metabolism of ketamine primarily occurs in the liver, where it undergoes biotransformation via cytochrome P450 enzymes, specifically CYP2B6, CYP2A6, and CYP3A4. These enzymes convert (R, S)-ketamine into its primary metabolites, norketamine (NK) and hydroxynorketamine (HNK) (Romagnoli et al., 2017) (Fig. 2). The elimination half-life of ketamine ranges from 2 to 4 h in humans, depending on the route of administration (IV, intramuscular, or oral). Ketamine can be detected in the blood for up to 24 h after administration, influenced by factors such as metabolism rate, dosage, and frequency of use (Clements et al. 1982). In rodent models, the half-life of ketamine is shorter, typically between 1 and 2 h, due to their higher metabolism rates. rapid metabolism leads to faster expulsion of ketamine, with detection in the blood for up to 6 to 12 h after administration (Table 2).

**Table 2:** Timeline for the short-term effects of ketamine (Grant et al. 1981; Yanagihara et al. 2003)

Human				
* Route of Administration*	*Onset*	*Peak Effects*	*Duration*	*Study*
Sublingual/Buccal	15–20 min	45–90 min	2–4 h (acute effects), up to 6 h (mild effects)	
Oral	20–30 min	1–2 h	3–4 h (acute effects), up to 6–8 h (mild effects)	
Intranasal (IN)	5–15 min	20–60 min	45–120 min (acute effects), up to 2–4 h (mild effects)	
Subcutaneous (SC)	5–10 min	15–30 min	30–90 min (acute effects), up to 2–4 h (mild effects)	
Intramuscular (IM)	2–10 min	15–30 min	1–2 h (acute effects), up to 4–6 h (mild effects)	
Intravenous (IV)	1–5 min	5–15 min	30–60 min (acute effects), up to 1–2 h (mild effects)	(Woelfer et al. 2020)
Rodent				
* Route of Administration*	*Onset*	*Peak Effects*	*Duration*	*Study*
Sublingual/Buccal	5–10 min	20–40 min	1–2 h (acute effects), up to 3 h (mild effects)	
Oral	10–20 min	30–60 min	1–2 h (acute effects), up to 4 h (mild effects)	(Ju et al. 2017; Viana et al. 2020)
Intranasal (IN)	5–10 min	15–30 min	30–60 min (acute effects), up to 2 h (mild effects)	
Subcutaneous (SC)	5–10 min	10–30 min	30–90 min (acute effects), up to 2–4 h (mild effects)	
Intramuscular (IM)	2–5 min	10–20 min	30–60 min (acute effects), up to 2 h (mild effects)	
Intravenous (IV)	1–2 min	3–10 min	15–30 min (acute effects), up to 1 h (mild effects)	(Choi et al. 2017; Radford et al. 2018, 2020; Zhang et al. 2019)
Intraperitoneal (IP)	5–10 min	10–20 min	30–90 min (acute effects), up to 2–4 h (mild effects	(Asim et al. 2020; Donahue et al. 2014; Duclot et al. 2016; Girgenti et al. 2017; Hou et al. 2018; Hu et al. 2023; Ito et al. 2015; Lee et al. 2019; Li et al. 2017; Ma et al. 2022a; Ma, Zhang, Ma et al. 2022a, b; Paredes et al. 2022; Ryan et al. 2022; Sala et al. 2022; Sun et al. 2022; Weckmann et al. 2019; Yang et al. 2022; Yang et al. 2014; Zhang et al. 2015)
Intracerebroventricular (ICV)	1–2 min	3–10 min	15–30 min (acute effects), up to 1–6 h (mild effects)	(Gou et al. 2023; Li et al. 2022; Xu et al. 2023)

The metabolism of ketamine in rodents quickly produces active metabolites such as NK and HNK, which play a significant role in its antidepressant properties. These metabolites are known to boost synaptic plasticity and promote neurogenesis in critical brain areas such as the HPC and PFC, which are essential for mood and cognitive functions (Schwenk et al. 2021). Similarly, in humans, ketamine undergoes extensive liver metabolism, leading to metabolites that prolong its therapeutic effects beyond the initial administration. Comparing the metabolic processes in rodents and humans shows a consistent pattern where ketamine’s immediate effects are succeeded by long-lasting neuroplastic changes induced by its metabolites (Wang et al. 2018). This understanding is crucial for optimising dosing strategies and enhancing the therapeutic outcomes of ketamine.

### Ketamine titration

The dosage of ketamine significantly affects its duration and intensity in the body. Higher doses lead to extended effects and longer detection periods (Schep et al. 2023). Frequent ketamine use results in its accumulation, altering its pharmacokinetics and expanding the detection window (Morgan et al. 2010). The route of administration also plays a critical role in ketamine’s absorption, metabolism, and elimination rates (Schwenk et al. 2021). For example, IV administration causes a rapid onset with a brief duration, while oral administration has a slower onset and prolonged effects due to first-pass metabolism in the liver (Bell and Kalso 2018). These factors collectively influence ketamine’s overall impact on both human and animal subjects (Table 3).

**Table 3:** Pharmacokinetic profiles of (R, S)-ketamine in rodent and humans (Chong et al. 2009; Yanagihara et al. 2003)

Route of Administration	Dosage (mg/kg)		Bioavailability		Tmax (min)	
	*Human*	*Rodent*	*Human*	*Rodent*	*Human*	*Rodent*
Intravenous (IV)	1.0–4.5	5–20	100%	100%	3	1–3
Intramuscular (IM)	6.5–13	20–50	93%	90–98%	5–10	5–15
Oral	0.25–0.5	10–30	17–29%	10–20%	30	20–40
Sublingual	10–25	15–40	24–30%	20–35%	30–45	15–30
Intranasal	0.5–1.0	10–25	8–45%	30–50%	10–20	5–15

This table reflects the general pharmacokinetic profiles for (R, S)-ketamine in human and mouse models, which are subject to variations based on specific experimental conditions and individual differences. Tmax = time to maximum concentration within the bloodstream

### The effect of ketamine on human brain regions

Understanding and enhancing ketamine’s therapeutic potential for PTSD requires identifying the specific brain regions it affects. Known for its rapid antidepressant effects, ketamine influences several key areas involved in emotion regulation, memory, and stress response. The PFC, critical for decision-making and executive processing, often shows impaired function in PTSD patients. Ketamine’s impact on the PFC may directly improve these cognitive functions (Fremont et al. 2023).

The HPC, essential for memory formation, typically exhibits reduced volume and aberrant activity in PTSD. Ketamine may act through neuroplastic changes within various substructures of the HPC, namely the dentate gyrus and CA3, to reverse some of the observed memory deficits. Additionally, the AMY, which processes fear and is critical in regulating emotional responses, tends to be hyperactive in PTSD. Ketamine’s effects on the AMY could help mitigate exaggerated fear responses (Kutlu et al. 2016).

Research also highlights other regions such as the anterior cingulate cortex (ACC), the bed nucleus of the stria terminalis (BNST), and the ventral tegmental area (VTA), which are all implicated in the regulation of stress and reward. By identifying these regions as therapeutic targets, researchers can better understand how ketamine alleviates PTSD symptoms, potentially leading to new effective treatments (Albuquerque et al. 2022).

Understanding these specific brain mechanisms not only aids in optimising dosing strategies but also helps in developing adjunctive therapies to enhance ketamine’s efficacy​ (Table 4).

**Table 4:** Human brain regions of interest in PTSD research

Brain Region	Subregion	Acronym	Structure	Function	Effect of PTSD
Prefrontal Cortex		PFC	Frontal lobe, anterior part of the brain.	Decision making, emotional regulation, executive functions	Reduced volume, impaired decision making, and emotional regulation.(Alexandra Kredlow et al. 2022)
	Medial Prefrontal Cortex	mPFC	Frontal lobe, medial aspect of the prefrontal cortex.	Regulates amygdala activity, involved in emotional and fear extinction	Decreased activity, leading to impaired regulation of fear responses.(Alexandra Kredlow et al. 2022)
	Anterior Cingulate Cortex	ACC	Anterior aspect of the cingulate cortex, surrounding the corpus callosum.	Cognitive functions, emotion regulation, and error detection	Reduced volume and function, leading to difficulties in emotion regulation and increased anxiety.(Tanaka et al. 2019)
Hippocampus		HPC	Medial temporal lobe, within the limbic system.	Memory formation and retrieval	Reduced volume, impaired memory function, and contextual fear processing.(McEwen et al. 2016)
	Dentate Gyrus	DG	Part of the hippocampal formation, adjacent to CA3 region.	Formation of new episodic memories, pattern separation	Reduced neurogenesis, impaired memory processing, and increased susceptibility to stress.(Dirven et al. 2022)
	CA1 Region	CA1	Part of the hippocampus, extends into the subiculum.	Long-term potentiation, memory encoding and retrieval	Altered synaptic plasticity, impaired spatial memory, and learning deficits.(Kozlovsky et al. 2007)
	CA3 Region	CA3	Part of the hippocampus, connected to DG and CA1 regions.	Memory retrieval, pattern completion, and spatial memory	Increased excitability, disrupted memory recall, and susceptibility to stress-induced damage.(Skorzewska et al. 2020)
Amygdala		AMY	Anterior aspect of the temporal lobe, within the limbic system.	Emotional processing and fear response.	Hyperactivity, exaggerated fear response, and emotional dysregulation.(McEwen et al. 2016)
	Basolateral Amygdala	BLA	Lateral part of the amygdala, within the temporal lobe.	Associated with sensory information of emotional responses.	Hyperactivity, contributing to exaggerated emotional responses and fear conditioning.(Kirby et al. 2012)
Nucleus Accumbens		NAc	Basal forebrain, near the septum and olfactory tubercle.	Reward processing and motivation.	Altered reward processing, reduced motivation, and anhedonia.(Yu et al. 2022)
Locus Coeruleus		LC	Brainstem, near the fourth ventricle.	Stress response, arousal, and norepinephrine release.	Increased activity, contributing to hyperarousal and heightened stress responses.(George et al. 2013)
Ventral Tegmental Area		VTA	Midbrain, near the substantia nigra.	Dopamine production, reward and motivation.	Altered dopamine signalling, contributing to anhedonia and changes in reward processing.(Ning et al. 2022)
Hypothalamic-Pituitary-Adrenal Axis		HPA	Interaction between the hypothalamus, pituitary gland, and adrenal glands.	Regulation of stress response and secretion of cortisol.	Dysregulation leads to chronic stress, altered cortisol levels, and impaired stress response mechanisms.(Dunlop and Wong 2019)
Dorsal Striatum		DS	Part of the basal ganglia, includes the caudate nucleus and putamen.	Motor control, habit formation and reward processing.	Altered reward processing and habit formation, contributing to maladaptive behaviours.(Alexandra Kredlow et al. 2022)
Periaqueductal Gray		PAG	Midbrain, surrounding the cerebral aqueduct.	Pain modulation, defensive behaviour, and autonomic function.	Increased activity, contributing to heightened pain perception and defensive behaviours.(Zhang et al. 2024)

### Existing understanding on the immediate and sustained effects of ketamine

Research involving both rodents and humans indicates that an acute ketamine infusion can lead to a rapid and substantial reduction in PTSD symptoms (Hashimoto et al. 2020). The immediate effects of ketamine are defined as those that occur while the drug is still active in the body. In rodent models, this effect is typically observed within the first 12 h post-infusion, whereas in human studies, the effect is generally assessed within the first 24 h (Domino et al. 1982; Liao et al. 2016).

In rodent models, acute ketamine administration can lead to a reduction in behavioural despair and anxiety-like behaviours within hours (Aikawa et al. 2020). This rapid effect has been attributed to ketamine’s action on the NMDA receptor, leading to increased synaptic plasticity and enhanced connectivity in brain regions associated with mood regulation, such as the PFC and HPC (Feder et al. 2020).

Similarly, in IV human clinical trials, a single infusion of ketamine has been observed to produce a marked decrease in PTSD symptoms, including reductions in intrusive thoughts, hyperarousal, and avoidance behaviours. These effects often emerge within hours to days post-infusion, providing a fast-acting alternative to traditional antidepressants, which typically take 4–6 weeks before clinical improvement takes place (Yermus et al. 2023).

Long-term effects of ketamine infusion present a more complex and nuanced picture in both rodent and human studies. In rodents, repeated ketamine administration has been linked to sustained antidepressant effects, with improvements in behaviour persisting for weeks (Berman et al. 2000). Chronic exposure to ketamine also leads to alterations in gene expression and synaptic function, which may underlie both the therapeutic benefits and potential risks (Dai et al. 2022). Ketamine has been shown to alleviate PTSD-like symptoms, such as cognitive impairments and anxiety-like behaviours, through various mechanisms (Raut et al. 2022). However, concerns have been raised about potential side effects and increased risk of substance abuse with prolonged use (Niesters et al. 2014). These findings collectively highlight the translational potential of ketamine from rodent models to human clinical applications, emphasising its multifaceted role in modulating neurobiological and inflammatory processes associated with PTSD.

This systematic review synthesises the current research findings on these molecular interactions and evaluates their immediate and sustained effects on PTSD treatment outcomes. By integrating data from diverse research methodologies, including longitudinal studies, randomised controlled trials, and meta-analyses, this review endeavours to clarify the complex mechanisms by which ketamine exerts its effects and maintains its efficacy over time. By understanding these mechanisms, the aim is to provide a molecular basis for the clinical efficacy of ketamine crucial for optimising treatment protocols and improving outcomes for PTSD patients as a step towards precision medicine.

## Methods

Using Preferred Reporting Items for Systematic Review and Meta-Analysis (PRISMA) guidelines, the search for this systematic review was conducted in August 2024 and registered on PROSPERO (ID: CRD42024582874). Primary databases searched included PubMed, Web of Science and Scopus, Secondary databases searched included Google Scholar, PsycINFO and SciSpace.

Search terms and their variations were “ketamine”, “PTSD”, “post-traumatic stress disorder”, “posttraumatic stress disorder”, “molecular mechanisms”, “biochemical pathways”, “cellular mechanisms”, “neural pathways”, “short-term effects”, “acute effects”, “long-term effects” and “chronic effects”.

The Boolean search string resulted in the following.

Basic search: (“ketamine” AND “PTSD” AND “molecular mechanisms”)

Differentiating Short-term and Long-term Effects: (“ketamine” AND “PTSD” AND (“short-term effects” OR “acute effects”) AND “molecular mechanisms”), (“ketamine” AND “PTSD” AND (“long-term effects” OR “chronic effects”) AND “molecular mechanisms”)

The search strategy was reviewed by all authors, and reports were independently screened by two investigators (A.V.K. and N.J.W) using the Covidence systematic review software to manage, track, and ensure proper filtering and sampling. Title and abstract screening, as well as full-text eligibility assessments, were conducted independently by the same two investigators. For quality assessment, the Cochrane Risk of Bias tool was employed to evaluate each study.

Eligible studies included were peer-reviewed and published research investigating the molecular mechanisms of ketamine’s effects on PTSD, examining short-term (immediate) and/or long-term (sustained) effects of ketamine on human and animal models and included: original research articles including clinical trials, observational studies, and preclinical studies; review articles that synthesise previous research on the molecular mechanisms of ketamine’s effects on PTSD; studies involving participants diagnosed with PTSD according to recognised diagnostic criteria (e.g., DSM-V or ICD-10); studies where ketamine was administered and studied as the primary intervention; studies that included clear outcome measures related to molecular and cellular mechanisms (e.g., changes in neurotransmitter levels, receptor modulations, gene expression); studies published within the last 20 years (to ensure the relevance and recency of the molecular data); and studies published in English. Data extracted for analysis included study design, sample size, route of administration or exposure to ketamine, measures of improvement or deterioration, time span for improvement or deterioration, main molecular and neurobiological results, findings and limitations.

Exclusion criteria included studies utilising confounding pharmacological interventions where results were not clear, non-PTSD recognised criteria, non-PTSD animal stress models, non-biological outcomes, conference materials, case-studies, general narrative and systematic reviews (not focused on molecular mechanisms) and studies where participants had comorbid disorders.

**Fig. 3:**
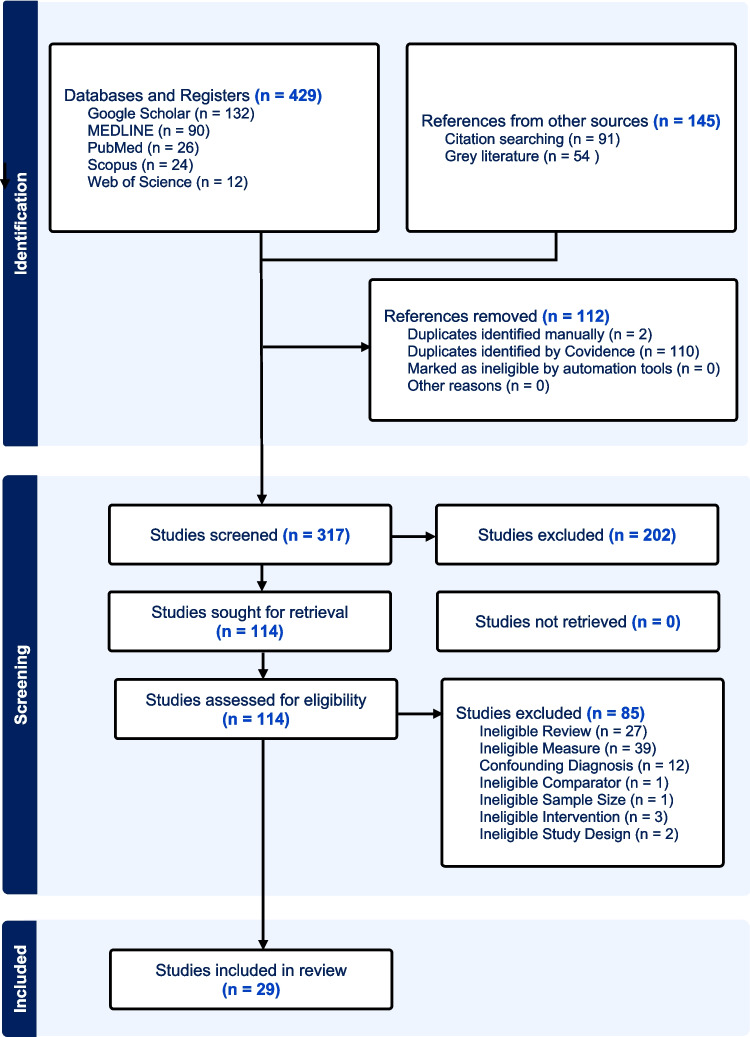
Schematic view of PRISMA methods utilised in the systematic review

The database and register search along with the citation and grey literature searching yielded 429 reports. The screening process removed 112 duplicated and led to an exclusion of 317 reports for reasons such as manuscripts only focusing on clinical outcomes, confounding diagnoses and reporting ineligible results. The final 29 articles focused on the molecular mechanisms of short- and long-term ketamine response on PTSD. These papers were examined and passed consensus criteria for the Cochrane risk of bias analysis (refer to Supplementary Material 1). The result from this analysis is included in the Prisma Workflow (Fig. 3).

## Results

### Study characteristics

Of the 29 studies assessed (Table 5) 16 utilised rats (Choi et al. 2017; Girgenti et al. 2017; Gou et al. 2023; Hou et al. 2018; Hu et al. 2023; Lee et al. 2019; Li et al. 2022; Paredes et al. 2022; Radford et al. 2020; Sala et al. 2022; Sun et al. 2022; Yang et al. 2014, 2022; Zhang et al. 2015, 2019), 12 were mouse models (Asim et al. 2020; Donahue et al. 2014; Duclot et al. 2016; Ito et al. 2015; Li et al. 2017; Ma et al. 2022a; Ma, Zhang, Ma et al. 2022a, b; Ryan et al. 2022; Sun et al. 2022; Viana et al. 2020; Weckmann et al. 2019; Xu et al. 2023) and one employed humans (Woelfer et al. 2020). Apart from the human study, each rodent model applied a combination of stressor environments to elicit a stress response which mimicked a particular aspect of PTSD including fear memory formation, fear conditioning and renewal, and fear memory recall.

**Table 5:** Immediate and sustained molecular effects of ketamine on PTSD in preclinical studies

Article	Year	Ketamine	Comp. Drug	Type	Dose	Admin	Methods	Model	Stress Model	< 24 h	> 24 h	Results
(2R, 6R)-hydroxynorketamine acts through GluA1-induced synaptic plasticity to alleviate PTSD-like effects in rat models(Li et al. 2022)	2021	HN-KET*		ICBV*	50mM	AC*	Protein levels of GluA1, BDNF, and PSD-95 were analysed using western blotting and immunofluorescence. Synaptic ultrastructure of the prefrontal cortex was observed using transmission electron microscopy.	Rat*	SPS&FS model included OFT, EMP, FST and FBT.	GluA1, BDNF, and PSD-95 protein expression in PFC increased. GluA1-BDNF signalling pathway activated in PFC. Glutamatergic neurotransmission in PFC improved.		(2R,6R)-HNK increased GluA1, BDNF, and PSD-95 protein expression and reversed synaptic ultrastructure changes induced by SPS&FS.
(2R, 6R)-hydroxynorketamine improves PTSD-associated behaviours and structural plasticity via modulating BDNF-mTOR signalling in the Nac(Gou et al. 2023)	2022	HN-KET	Saline	ICBV	10, 50, 100mM	AC	Intervention included a 7-day recuperation, followed by the intra-nuclear administration of (2R,6R)-HNK into the Nac, and behavioural tests conducted seven days post-treatment.	Rat (*n* = 60/ Male)	SPS&FS model conducted post-intervention included OFT and SAT to evaluate social anxiety levels.		Re-regulated BDNF and mTOR synaptic plasticity in the Nac	(2R,6R)-HNK improved exploration, depression-linked behaviour, and synaptic ultrastructure in rats and restored protein levels and synaptic morphology in rats.
(2R, 6R)-hydroxynorketamine targeting the BLA regulates fear memory(Xu et al. 2023)	2023	HN-KET		ICBV	30 mg/kg	AC	HNK was microinfused into the BLA 30 min after recall, followed by extinction trials 30 min later. Extracellular electrophysiological recording for synaptic transmission and plasticity assessment. Micro infusion of (2R,6R)-HNK into the BLA for analysis	Mouse*	AFC model investigating fear memory formation and extinction.	Increases in c-Fos-positive cells in BLA within 24 h increase in BLA synaptic transmission and plasticity.		(2R,6R)-HNK enhanced BLA synaptic transmission and plasticity, reduced retrieval of recent and remote fear memories, and inhibited spontaneous recovery of remote fear memory.
A key role of miR-132-5p in the PFC for persistent prophylactic actions of (R)-ketamine in mice(Ma et al. 2022)	2021	(R)-KET		IP*	10 mg/kg	Prop. AC*	RNA sequencing analysis of PFC for miR-132-5p. AgomiR-132-5p administration to study depression-like behaviours.	Mouse	CRS model used for 7 days. The behavioural tests conducted after the intervention included FST and SPT to assess depression-like behaviours.		miR-132-5p role in PFC for sustained prophylactic effects of (R)-ketamine. (R)-ketamine attenuated altered expression of miR-132-5p and regulated genes. (R)-ketamine improved reduced levels of BDNF and TGF-b1 in PFC. MeCP2 regulates BDNF expression in the PFC.	Inhibition of miR-132-5p contributes to persistent prophylactic effects of (R)-ketamine.
Acute ketamine facilitates fear memory extinction in a rat model of PTSD along with restoring glutamatergic alterations and dendritic atrophy in the PFC(Sala et al. 2022)	2021	(R, S)-KET*	Desipramine	IP	10 mg/kg	Prop. AC	Measurement of glutamate release in naive animals treated with ketamine. Experiments on neuronal cultures. Electrophysiological measurements. Golgi-cox analysis.	Rat	FS, SPT and NSFT were used. Fear extinction was also preformed 1–4 days after test and 3–7 days after test.	Ketamine blocked stress-induce glutamate release and rescued dendritic retraction in the prelimbic prefrontal cortex, stabilises glutamate dysfunction within 24 h post-stress exposure.	Miniature excitatory PSCs were recorded in pyramidal neurons within PL-PFC recorded 24 h after acute stress.	Ketamine blocked stress-induced glutamate release in the PFC and restored glutamate release, dendritic atrophy, and fear extinction behaviours.
Adjunct treatment with ketamine enhances the therapeutic effects of extinction learning after chronic unpredictable stress(Paredes et al. 2022)	2022	(R, S)-KET		IP	10 mg/kg	AC		Rat (*n* = 354 Male)	CUS model and attentional set-shifting test were used in the study including fear conditioning and extinction protocols. Acute stressors applied daily for 14 days for males, 21 days for females. Extinction protocols tested with different tones in male and female rats.	Ketamine did not enhance MDT-evoked LFPs in the mPFC. Ketamine and extinction share BDNF-associated plasticity mechanisms.		Adjunct ketamine enhanced extinction efficacy, reversing stress-induced cognitive deficits combined with a shortened, sub-effective extinction protocol fully reversed stress-induced cognitive set-shifting deficits in both male and female rats.
Anxiolytic effects of ketamine in animal models of PTSD(Zhang et al. 2015)	2015	(R, S)-KET	Sertraline	IP	2.5 mg/kg	CH*	HPC was isolated for Western blotting detection. The protein concentration was determined by a BCA assay.	Rat	TDS model used included FS to induce PTSD-like symptoms. The behavioural tests conducted after the intervention included the OFT, EPM and the contextual fear paradigm.	Ketamine normalised decreased BDNF levels in the HPC post-TDS.	Ketamine altered BDNF mRNA expression in the rat brain. BDNF protein levels were measured in the HPC post-ketamine treatment.	Ketamine normalised decreased BDNF levels in the HPC post-TDS. Sertraline also ameliorated behavioural deficits in the animal models. Ketamine showed anti-PTSD effects mediated by BDNF signalling in the HPC.
Applying ketamine to alleviate the PTSD-like effects by regulating the HCN1-related BDNF(Hou et al. 2018)	2018	(R, S)-KET		IP	20 mg/kg	AC		Rat (*n* = 56)	SPS&FS procedure of PTSD. Post behavioural tests conducted included OFT, EPM, and FST to assess locomotor behaviour, exploratory behaviour, and immobility time.	Ketamine increased BDNF, decreased HCN1 in PFC within 24 h. Negative correlation between BDNF and HCN1 in the PFC.		Ketamine alleviated PTSD-like behaviours by regulating BDNF and HCN1 levels. Ketamine increased locomotor behaviour and exploratory behaviour in rats.
Association between intravenous ketamine-induced stress hormone levels and long-term fear memory renewal in Sprague-Dawley rats(Radford et al. 2020)	2021	(R, S)-KET		IV*	10 mg/kg (2 h)	AC	Tail flick test for antinociception measurement. CORT and PROG levels measured using ELISA kits.	Rat	The behavioural tests conducted included fear extinction acquisition through AFC on Day 7, fear extinction retrieval on Day 8, and fear renewal on Day 9, following the ketamine infusion.	Increases in CORT hormone levels within 24 h indicating ketamine stimulates HPA. Increases in PROG as a precursor to CORT suggesting a release from adrenal gland.		Ketamine infusion led to sedation, antinociception, and HPA axis stimulation. No alteration in long-term fear memory with ketamine infusion. Elevated corticosterone and progesterone levels correlated with sedation and memory renewal.
Effects of ketamine on levels of inflammatory cytokines il-6, il-1beta, and TNF-α in the HPC of mice following acute or chronic administration(Li et al. 2017)	2017	(R, S)-KET		IP	10–80 mg/kg CH(30–60 mg/kg)	AC/CH	Acute and chronic ketamine administration models in C57BL/6 mice. Behavioural tests, Western blot, qRT-PCR, and immunohistochemistry for analysis. Chronic experiment with a 6-month daily i.p. injection of ketamine. qRT-PCR method for RNA isolation and cDNA synthesis.	Mouse (*n* = 180)	Chronic stress-induced depression model used for ketamine research performed 3 h after i.p. injection, these included the OFT and EMP.	Ketamine increased IL-6 and IL-1β levels in the hippocampus. TNF-α expression varied in the hippocampus based on ketamine administration. TNF-α showed bi-directional regulation based on ketamine dose and duration.	6 months post Ketamine increased the levels of IL-6, IL-1β, and decreased the TNF-α levels in the hippocampus. Chronic ketamine administration led to spatial memory deficits in mice.	Acute administration increased the levels of IL-6 and IL-8 with varied expression in TNF-α. Chronic ketamine increased IL-6 and IL-1β levels, reduced TNF-α expression. 60 mg/kg ketamine induced spatial memory deficit and reduced anxiety. Acute ketamine had no effect on anxiety or spatial memory.
Effects of striatal ΔFosB overexpression and ketamine on social defeat stress-induced anhedonia in mice(Donahue et al. 2014)	2015	(R, S)-KET		IP	20 mg/kg	AC	Intracranial self-stimulation (ICSS) to measure reward function in mice. Ketamine administration to assess effects on social interaction and reward.	Mouse	Chronic SDS model was used and triggers persistent anhedonia and stress resilience in mice.		ΔFosB overexpression in striatum mediates stress resilience to CSDS. CSDS triggers persistent anhedonia, confirmed by ΔFosB overexpression. ΔFosB -ON mice show resilience to CSDS-induced anhedonia	CSDS increased ICSS thresholds, indicating anhedonia, attenuated by ΔFosB overexpression. Ketamine did not attenuate anhedonia in the ICSStest.
Effects of subanesthetic intravenous ketamine infusion on corticosterone and brain-derived neurotrophic factor in the plasma of male sprague-dawley rats(Radford et al. 2018)	2018	(R, S)-KET	Saline (Control)	IV	5 mg/kg 20 mg/kg (2 h)	AC	The intervention included blood sampling at consistent times, 2 h after fear conditioning, and 4 h after fear conditioning. Blood sampling for corticosterone and BDNF analysis.	Rat	AFC model used	Intermediate CORT levels enhance fear memory consolidation in the amygdala. Dose-dependently increased plasma corticosterone levels. Ketamine at 20 mg/kg/h significantly reduced plasma BDNF 2 h after the ketamine infusion.		Ketamine infusion increased plasma CORT and reduced plasma BDNF concentrations. IV ketamine post-fear conditioning affected stress and fear memory biomarkers.
Effects of subanesthetic intravenous ketamine infusion on neuroplasticity-related proteins in the prefrontal cortex, amygdala, and hippocampus of Sprague-Dawley rats(Zhang et al. 2019)	2019	(R, S)-KET		IV	40 mg/kg 10 mg/kg (2 h)	AC	Western blot analysis conducted on brain tissue post-infusion. Brain regions dissected for protein quantification using specific antibodies.	Rat (*n* = 18 Male, *n* = 10 Female)	AFC model used (0.8 mA, 0.5 s)	Measured 2 h after a 2-hour Ketamine infusion affects c-Fos, BDNF, and pERK in brain regions. IV ketamine infusion induces dose-dependent and region-specific protein level changes.		High dose ketamine increased c-Fos in mPFC and amygdala as well as pERK in the mPFC and HPC. Low dose ketamine raised BDNF in AMY, decreased pERK in mPFC.
Ketamine accelerates fear extinction via mTORC1 signalling(Girgenti et al. 2017)		(R, S)-KET	Rapamycin, NBQX, saline	IP	10 mg/kg	AC	mTORC1 inhibitor rapamycin infusion into the mPFC. Western blotting for protein analysis post behavioural testing. Brain tissue collected 90 min after extinction recall test on day 2. Western blotting performed with specific primary antibodies for protein analysis.	Rat	AFC model used for fear conditioning extinction and renewal. Ketamine administration after AFC, followed by extinction training. Extinction training over three days and assessment of spontaneous recovery and fear renewal one week later.		Ketamine and extinction exposure increased mTORC1 levels in the mPFC, which is involved in extinction learning and retrieval. Infusion of the selective mTORC1 inhibitor rapamycin into the mPFC blocked ketamine’s effects on extinction. Ketamine plus extinction also increased cFos in the mPFC and administering a glutamate-AMPA receptor antagonist blocked ketamine’s effects.	Ketamine enhanced extinction recall and reduced freezing behaviour in rats. mTORC1 activation in the mPFC mediated ketamine’s effects on extinction. Ketamine improved fear extinction and reduced fear renewal in rats.
Ketamine alleviates fear generalisation through GluN2B-BDNF signalling in mice(Asim et al. 2020)	2020	(R, S)-KET	Ifenprodil, ANA-12	IP	30 mg/kg	AC	Mice were sacrificed 1–2 weeks post behaviour test and samples were immunoblotted.	Mouse	AFC model used for fear conditioning and fear memory recall.		Ketamine reverses BDNF and GluN2B levels in BLA and IL-PFC. ANA-12 blocks ketamine’s effect on fear generalisation in mice.	The fear-generalised mice showed a lower level of BDNF and a higher level of GluN2B protein in the BLA and IL-PFC, and this was reversed by a single administration of ketamine. Ketamine 22 h post-conditioning reduced fear generalisation, dose-dependent, lasting 2 weeks. Ketamine increased BDNF, decreased GluN2B, reversed fear generalisation in mice.
Ketamine alleviates fear memory and spatial cognition deficits in PTSD rat model via BDNF signalling pathway of HPC and AMY.(Sun et al. 2022)	2022	(R, S)-KET		IP	5–20 mg/kg (Optimal dose: 10–15 mg/kg)	AC	Blood and brain tissue were collected on day 22	Rat	SPS&FS model used. Rats were left undisturbed in their home cages for 14 days. On day 15, the drugs were administered intraperitoneally. The behavioural tests conducted included FBT 24 h after drug treatment and the Morris Water Maze (MWM) during day 17 to day 21.		BDNF and PSD-95 signalling changes in HPC and AMY. Ketamine reversed cognitive impairment and molecular changes in the HPC.	Ketamine at 10–15 mg/kg improved cognitive function and molecular changes. Ketamine reduced freezing time and improved spatial memory in rats.
Ketamine ameliorates severe traumatic event-induced antidepressant-resistant depression in a rat model through ERK activation(Lee et al. 2019)	2019	(R, S)-KET	Fluoxetine	IP	10 mg/kg	Prop. AC	Western blot analysis was performed	Rat	FBT included FS, NSFT, FST and Tail suspension test.		ERK phosphorylation decreased in AMY and PFC. GluA1 and PSD 95 immunocontents altered in AMY and PFC. Glutamate-related abnormalities in AMY and PFC normalised by ketamine. ERK activation observed in the rat model over 24 h.	Severe stress led to SSRI-resistant depression in rats. Ketamine reversed traumatic stress-induced changes in synaptic proteins.
Ketamine induces BDNF expression via phosphorylation of histone deacetylase 5 in rats(Choi et al. 2017)	2016	(R, S)-KET		IV	10 mg/kg	AC	Lentivirus for HDAC5 knockdown, for in vivo injections ranging from 8 to 10 million to 20 million. Rat HPC neuron preparation and processing. Quantitative real-time RT-PCR for gene expression analysis. Western blot analysis for protein expression. Luciferase reporter assay for BDNF IV promoter activity. Immunohistochemistry for immunofluorescent labelling.	Rat	A 28-day exposure to CUS, day 7 - lentivirus injection, day 34 - ketamine injected. The behavioural tests included the NSFT, SPT, FST and the learned helplessness test to assess the effects of ketamine on behaviour. Stressors applied twice daily for 28 days.	Ketamine increases BDNF mRNA levels within 30 min of injection. BDNF mRNA peaks at 6 h and remains elevated for 24 h. Ketamine induces BDNF exon IV mRNA significantly within 30 min.	BDNF levels in the HPC increase significantly 24 h post-injection. Ketamine induces BDNF expression via HDAC5 phosphorylation in HIP. BDNF promoter IV activity increases in response to ketamine treatment. HDAC5 knockdown blocks ketamine-induced BDNF expression in dentate gyrus.	The regulation of BDNF expression in neurons by ketamine via the phosphorylation of HDAC5 and suggest that induction of BDNF expression by ketamine may result from the suppression of the repressor activity of HDAC5.
Ketamine-induced changes in plasma BDNF levels are associated with the resting-state functional connectivity of the PFC(Woelfer et al. 2020)	2020	(R, S)-KET	Saline	IP	0.5 mg/kg	AC/CH (40 min)	Randomised, placebo-controlled study with 53 healthy participants. Investigated Resting-State Functional Connectivity (RSFC) changes post-ketamine infusion using 7 T-fMRI. Plasma samples were acquired 120 min post-infusion, and the third fMRI scan and blood withdrawal were done exactly 24 h after the baseline measurements. BDNF levels quantified using ELISA assay and whole-brain regression models.	Human (*n* = 80)	N/A	Higher BDNF levels at 2 and 24 h post ketamine BDNF changes are associated with RSFC decreases in specific brain regions.	BDNF changes were associated with RSFC decreases in specific brain regions. Ketamine group showed stronger RSFC decreases linked to BDNF increases. Plasma BDNF changes after 24 h affected RSFC in various brain regions. Ketamine group had stronger RSFC decreases in decreases from dmPFC to posterior cingulate cortex and vmPFC specific brain regions.	Higher BDNF levels post-ketamine infusion associated with RSFC changes. RSFC decreases from dmPFC to PCC and vmPFC after ketamine. BDNF dynamics post-ketamine linked to acute and 24 h RSFC changes.
Ketamine’s effects on the glutamatergic and GABAergic systems: A proteomics and metabolomics study in mice(Weckmann et al. 2019)	2019	(S)-KET	Saline	IP	3 mg/kg	AC	Targeted metabolomics and proteomics analysis with in vivo 15 N labelling. SRM-based metabolomics platform and quantitative MSMS for proteomics analysis. HPC metabolome and proteome analysed after ketamine treatment	Mouse	Forced swim test (FST), conducted after ketamine treatment was the performed at 2-, 14-, 24-, and 72-hours post-treatment.	HPC glutamate and glutamine levels significantly downregulated 14 h after infusion, two NMDAR metabolite modulators putrescine and serine were both elevated 2 h after infusion. Increased AMPAR subunit Gria2 protein levels observed 2 h after ketamine treatment. at 2 h and 24 h Myelin Based Protein (MBP) levels increased.	HPC GABA levels increased at 72 h, ketamine decreased GABAARa1 and AMPAR subunit Gria3 levels.	Ketamine altered GABA, glutamate, and glutamine levels in mice. Elevated levels of positive NMDAR modulators correlated with antidepressant-like effects.
MicroRNA expression profile and functional analysis reveal that miR-206 is a critical novel gene for the expression of BDNF induced by ketamine(Yang et al. 2014)	2013	(R, S)-KET		IP	15 mg/kg	CH (3 days)	miRNA expression profiling in rat hippocampus after ketamine treatment. qRT-PCR and Western Blot analysis were performed on the samples.	Rat (*n* = 24)	No stress model used.	miR-206 down-regulated in HPC within 12 h after ketamine treatment.	miR-206 down-regulated in rat HPC after ketamine treatment. Ketamine attenuated apoptosis induced by overexpression of miR-206. High miR-206 expression increased ICa and IA in HPC neurons.	BDNF is a target gene for mir-206, as ketamine attenuates mir-206, ketamine significantly increased BDNF expression in rat HPC.
Nuclear factor of activated T-cells 4 in the PFC is required for prophylactic actions of (R)-ketamine(Ma et al. 2022)	2022	(R)-KET	NFAT inhibitor, LPS treatment	IP	10 mg/kg	Prop. AC	Mice were treated with (R)-ketamine and NFAT inhibitors. Behavioural tests and gene expression analysis were conducted. Western blot analysis and statistical analysis were performed.	Mouse	Chronic SDS model and postpartum depression. The behavioural tests included OFT and FST 23 and 24 h after the injection of saline or LPS.		NFATc4 signalling in PFC contributes to prophylactic effects of (R)-ketamine. NFAT inhibitors showed prophylactic effects in LPS-treated mice. NFATc4 gene knockdown in mPFC elicits prophylactic effects in mice. (R)-ketamine attenuates NFATc4 gene expression in PFC of LPS-treated mice.	(R)-ketamine showed prophylactic effects on depression-like behaviour and systemic inflammation. NFATc4 signalling in the PFC contributes to the prophylactic effects. NFAT inhibitors and NFATc4 knockdown elicited similar prophylactic effects.
Observation of distressed conspecific as a model of emotional trauma generates silent synapses in the prefrontal-AMY pathway and enhances fear learning, but ketamine abolishes those effects(Ito et al. 2015)	2015	(R, S)-KET		IP	10 mg/kg	AC	Silent synapse detection: CV analysis and minimal-like stimulation methods. Recordings of AMPAR and NMDAR currents in neurons.	Mouse	OFM observer and demonstrator mice were separated, and demonstrator mice were administered FST. Fear conditioning model applied AFC. Enhanced passive avoidance (PA) learning when trained 24 h after exposure.		Higher AMPA/NMDA and lower silent synapses were observed in dmPFC and BLA. Ketamine abolished silent synapse formation in the prefrontal-AMY pathway. Observational fear exposure altered synaptic transmission in the dmPFC.	Enhanced passive avoidance learning observed after exposure. Ketamine administration prevented enhancement of PA learning and silent synapse formation. Emotional trauma generated silent synapses in the PFC-AMY pathway.
Prediction of individual differences in fear response by novelty seeking, and disruption of contextual fear memory reconsolidation by ketamine(Duclot et al. 2016)	2017	(R, S)-KET		IP	10 mg/kg	AC	Samples were collected 24 h after infusion. In situ hybridisation for Egr1 and BDNF mRNA levels analysis in the HPC, the mPFC and AMY.	Mouse	Fear conditioning model for NSFT and fear extinction included AFC. The timeline included reactivating contextual memory 10 days following the contextual memory test, and re-exposure 18 days after cue extinction.		Ketamine disrupts contextual fear reconsolidation over 24 h. Ketamine down-regulates Egr1 in the hippocampal CA1 area. Ketamine upregulates Bdnf mRNA in the prelimbic and infralimbic cortices.	Novelty seeking predicts fear response and extinction differences in rats. Ketamine disrupts contextual fear memory reconsolidation, reducing fear memory. HR rats show faster contextual fear extinction compared to LR rats.
Rapid and long-lasting antidepressant-like effects of ketamine and their relationship with the expression of brain enzymes, BDNF, and astrocytes(Viana et al. 2020)	2014	(R, S)-KET	Imipramine	Oral	2–10 mg/kg	AC	Immunohistochemical data calculated using ImageJ software for absorbance measurements. Antibodies for GSK-3, HDAC, BDNF, and GFAP immunohistochemical assays.	Mouse	FST used 30 min, 15 days, and 30 days after Ketamine administration.	Brain GSK-3 and HDAC immunoreactivities decreased in striatum, DG, CA1 CA3 and PFC. BDNF increased in PFC, DG, CA1, and CA3 areas.	GFAP and BDNF expression increased 30 days post-KET administration. PFC, DG, CA1, and CA3 showed changes in GSK-3, HDAC, BDNF, and GFAP.	Ketamine showed rapid and long-lasting antidepressant-like effects in mice. Decreases in GSK-3 and HDAC immunoreactivities were observed in various brain areas. BDNF and GFAP expressions increased in the brain 30 days post-administration.
S-ketamine pre-treatment alleviates anxiety-like behaviours and mechanical allodynia and blocks the pro-inflammatory response in striatum and periaqueductal gray from a PTSD model(Yang et al. 2022)	2021	(S)-KET		IP	5 mg/kg	AC (24 h after exposure)	SPS model for PTSD, S-ketamine treatment, behavioural tests, brain region analysis. Western blot for protein levels, immunofluorescence staining for microglia activation. Antibody incubation, secondary antibody treatment, confocal microscopy, statistical analysis.	Rat	Single-prolonged stress (SPS) exposure. Paw withdrawal mechanical threshold was measured 2 days prior to infusion, 1,3,5,7,10,14 and 21 days post infusion. OFT and EPM were performed at 7 and 14 days after S-ketamine treatment in the SPS-exposed rats.	S-Ketamine reduced microglia activation and pro-inflammatory cytokines in dorsal striatum. PAG showed microglia activation and upregulation of pro-inflammatory cytokines.	TNF-α, IL-1β, p-NFkB, and NF-kB were upregulated in the dorsal striatum and PAG, rather than ACC and PFC. S-ketamine reduced microglia activation and reversed TNF- α, IL-1β, p-NFkB and NF-kB pro-inflammatory cytokines in the dorsal striatum.	S-ketamine alleviated anxiety and mechanical allodynia in SPS-exposed rats. Reduced pro-inflammatory cytokines in dorsal striatum and PAG.
The combination of long-term ketamine and extinction training contributes to fear erasure by BDNF methylation(Ju et al. 2017)	2017	(R, S)-KET	Fluoxetine	Oral	AC(5,10,20 mg/kg 2 h) CH(0.625- 2.5 mg/kg 22 days post-trauma)	AC/CH	Real-time RT-PCR for mRNA levels in mPFC and HIP. Western blot analysis of mPFC and HIP was undertaken. DNA methylation analysis was performed.	Mouse (*n* = 165 Male)	Ketamine administration starting 1 h after the FS and short-term ketamine treatment 2 h prior to the extinction training on days 15 and 16 after FS. OFM and EPM tests on days 18 and 20, spontaneous recovery, and fear renewal tests on day 23.	Increased BDNF expression in mPFC and HPC after ketamine treatment. Hypo-methylation of BDNF exon IV observed after ketamine and extinction.	Long-term ketamine treatment (22Days) with extinction training normalised BDNF exon IV gene methylation in DNMT3a and DMNT3b and increased BDNF expression in mPFC and HPC.	Long-term ketamine with extinction training alleviated fear relapse in mice. Combination treatment induced erasure of conditioned fear by up-regulating BDNF.
The potential role of GSK-3β signalling pathway for amelioration actions of ketamine on the PTSD rodent model(Hu et al. 2023)	2023	(R, S)-KET	GSK-3β antagonist SB216763	IP	10 mg/kg	AC	SPS model simulation for PTSD-like symptoms. Ketamine and GSK-3β antagonist administration. Behavioural tests, qEEG, western blot, and qPCR for evaluations.	Rat	SPS model, including the immobilisation test, FST and diethyl ether. The behavioural tests conducted post-intervention included OFT and the EPM to evaluate stress-related behaviour.	Hypothalamus showed altered protein and gene expressions and protein levels of GSK-3β and GR within 24 h.	Ketamine reduced the protein levels of GSK-3β, GR, p-GSK-3β, and altered the ratio of p-GSK-3β to GSK-3β. Gene expression of GSK-3β, GR, BDNF, and FKBP5 decreased in the SPS-ketamine group compared to the SPS control group.	Ketamine improved anxiety-like behaviour in SPS-exposed rats. Ketamine reduced GSK-3β protein levels and altered gene expressions.
The role of BDNF in mediating the prophylactic effects of (R, S)-ketamine on fear generalisation and extinction(Ryan et al. 2022)	2021	(R, S)-KET		IP	30 mg/kg	Prop. AC	Behavioural testing on male adult WT and BDNF Val66Met mice Utilised fibre photometry to analyse neural activity in ventral HPC	Mouse	Behavioural model of fear generalisation in mice involving FS followed by four days of fear recall.	BDNF Val66Met mice showed fear generalisation within 24 h. Ventral HPC activity altered by prophylactic (R, S)-ketamine within 24 h.	Ventral HPC activity patterns mediated by BDNF altered by prophylactic (R, S)-ketamine treatment and reduces fear generalisation effect. BDNF Val66Met inhibited the R/S Ketamine effect on depression.	Prophylactic (R, S)-ketamine enhances fear discrimination in wild type mice. BDNF Val66Met mice resist protective effects of prophylactic (R, S)-ketamine. Ventral HPC activity altered by prophylactic (R, S)-ketamine in mice.

*AC* acute administration, *CH* chronic administration, *Prop.* prophylactic administration, *ICBV* intracerebroventricular, *IP* intraperitoneal, *IV*: intravenous, *(R, S)-KET* racemic ketamine, *HN-KET* hydroxynorketamine, Mouse*, Rat*: sample size not provided

The most commonly used behavioural model (in 7 studies) was the auditory fear conditioning model (AFC) where rodents learn to associate a neutral auditory stimulus, such as a tone, with an aversive stimulus, typically a mild foot shock (FS) (Girgenti et al. 2017; Ito et al. 2015; Radford et al. 2018, 2020; Xu et al. 2023; Zhang et al. 2019). This model is used to study the mechanisms of fear learning and memory by measuring conditioned responses, such as freezing behaviour, in response to the tone alone. The second most utilised stress model (in 6 studies) was the Single Prolonged Stress and foot shock (SPS&FS) model, used to induce PTSD-like symptoms in rodents by exposing them to a series of stressors, including restraint, forced swim, and ether anaesthesia, followed by a foot shock (Gou et al. 2023; Hou et al. 2018; Hu et al. 2023; Li et al. 2022; Teng et al. 2024; Yang et al. 2022). This model mimics the effects of traumatic stress and is used to study the neurobiological mechanisms and potential treatments for PTSD.

Ketamine administration varied dependent on the study’s design and objectives, with 19 studies focusing on acute ketamine administration (< 24 h) (Asim et al. 2020; Choi et al. 2017; Donahue et al. 2014; Duclot et al. 2016; Girgenti et al. 2017; Gou et al. 2023; Hou et al. 2018; Hu et al. 2023; Ito et al. 2015; Ju et al. 2017; Li et al. 2017, 2022; Paredes et al. 2022; Radford et al. 2020; Sun et al. 2022; Viana et al. 2020; Weckmann et al. 2019; Woelfer et al. 2020; Xu et al. 2023; Yang et al. 2022; Zhang et al. 2019), 5 studies incorporating prophylactic ketamine therapy (Lee et al. 2019; Ma et al. 2022a; Ma, Zhang, Ma et al. 2022a, b; Ryan et al. 2022; Sala et al. 2022), 3 studies combining both acute and chronic therapies for experimental comparison (Ju et al. 2017; Li et al. 2017; Woelfer et al. 2020), and 2 studies focused on chronic administration (> 24 h) outcomes (Yang et al. 2014; Zhang et al. 2015). All studies were performed within a one month time-frame with the exception of one mouse model which administrated chronic doses of ketamine over a six month period and is considered the only study on the long term- chronic effects of ketamine (Li et al. 2017).

The predominant form of ketamine utilised in the therapy was racemic (R, S)-ketamine, administered in 22 studies (Asim et al. 2020; Choi et al. 2017; Donahue et al. 2014; Duclot et al. 2016; Girgenti et al. 2017; Hou et al. 2018; Hu et al. 2023; Ito et al. 2015; Ju et al. 2017; Lee et al. 2019; Li et al. 2017; Paredes et al. 2022; Radford et al. 2018, 2020; Ryan et al. 2022; Sala et al. 2022; Sun et al. 2022; Viana et al. 2020; Woelfer et al. 2020; Yang et al. 2014; Zhang et al. 2015, 2019) alongside 3 studies that administered HNK (Gou et al. 2023; Li et al. 2022; Xu et al. 2023), 2 studies that administered R-ketamine (Ma et al. 2022; Ma, Zhang, Ma et al. 2022a, b), and 2 studies that administered S-ketamine (Weckmann et al. 2019; Yang et al. 2022).

Further, the dosage rates for ketamine varied dependent of the administration route and the study design. The dosage most frequently used was 10 mg/kg the mean dose was 21.67 mg/kg (SD = 25.13 mg/kg) with a range between 0.3 mg/kg-100 mg/kg **(**Table 5**).**

The administration type also determines the potency of the ketamine and its duration. Twenty studies administered ketamine intraperitoneally (Asim et al. 2020; Donahue et al. 2014; Duclot et al. 2016; Girgenti et al. 2017; Hou et al. 2018; Hu et al. 2023; Ito et al. 2015; Lee et al. 2019; Li et al. 2017; Ma et al. 2022a; Ma, Zhang, Ma et al. 2022a, b; Paredes et al. 2022; Ryan et al. 2022; Sala et al. 2022; Sun et al. 2022; Weckmann et al. 2019; Yang et al. 2014, 2022; Zhang et al. 2015), while 4 injected ketamine intravenously (Choi et al. 2017; Radford et al. 2018, 2020; Zhang et al. 2019), 3 through intracerebroventricular injection (Gou et al. 2023; Li et al. 2022; Xu et al. 2023) and 2 studies through oral dose (Ju et al. 2017; Viana et al. 2020) (Fig. 2).

Finally, 13 studies combined supplementary and control therapies to distinguish molecular responses to ketamine. These included:

- Desipramine (tricyclic antidepressant) - used in rodent models to study depression, anxiety, and neuropathic pain. Desipramine is often used to evaluate the efficacy of antidepressant treatments and to understand the mechanisms of depression (Sala et al. 2022).
- Fluoxetine (SSRI) - commonly used in rodent models to study depression, anxiety, and obsessive-compulsive behaviours. Fluoxetine helps in understanding the role of serotonin in mood regulation and the effects of chronic stress (Ju et al. 2017; Lee et al. 2019).
- Ifenprodil (NMDA receptor antagonist) - used to investigate the role of NMDA receptors in neuroprotection, neurodegeneration, and synaptic plasticity. Ifenprodil is often utilised in studies of depression, PTSD, and other neuropsychiatric disorders (Sun et al. 2022).
- ANA-12 (TrkB receptor antagonist) - utilised in rodent models to study the role of BDNF in neuroplasticity, depression, and anxiety. ANA-12 helps to elucidate the effects of blocking BDNF signalling in these conditions (Sun et al. 2022).
- Imipramine (tricyclic antidepressant) - used in rodent models to study depression and anxiety. Imipramine helps to evaluate antidepressant mechanisms and the efficacy of new treatments for mood disorders (Viana et al. 2020).
- NFAT Inhibitor (calcineurin inhibitor) - used to study immune responses and inflammation in various conditions, including neuroinflammatory and neurodegenerative diseases. NFAT inhibitors help understand the role of calcineurin-NFAT signalling in these processes (Ma et al. 2022).
- Rapamycin (mTOR Inhibitor) - used in rodent models to study ageing, cancer, and neurodegenerative diseases. Rapamycin helps in understanding the role of the mTOR pathway in cell growth, proliferation, and survival (Girgenti et al. 2017).
- NBQX (AMPA receptor antagonist) - utilised in studies to investigate the role of AMPA receptors in synaptic transmission, neuroprotection, and excitotoxicity. NBQX is used in research on stroke, epilepsy, and neurodegenerative diseases (Girgenti et al. 2017).
- Saline (control vehicle) - used as a placebo or control treatment in experiments to ensure that observed effects are due to the active drug and not the injection process or solution itself (Girgenti et al. 2017; Gou et al. 2023; Radford et al. 2018; Weckmann et al. 2019; Woelfer et al. 2020).
- Sertraline (SSRI) - used in rodent models to study depression, anxiety, and PTSD. Sertraline assists in understanding the role of serotonin in these conditions and evaluating the efficacy of antidepressant treatments (Zhang et al. 2015).

These drugs are widely used in preclinical research to investigate the mechanisms underlying various neuropsychiatric and neurodegenerative disorders, as well as to evaluate the efficacy and safety of potential treatments. Each drug serves a specific role in modelling human diseases in mice and contributes to the development of new therapeutic strategies.

### Prophylactic effects of ketamine treatment

This review found that an emerging area of research in rodent models has been the prophylactic use of ketamine to prevent the onset of PTSD. One mouse study testing fear generalisation, administered prophylactic (R, S)-ketamine 1 week prior to AFC and FS and found reduced fear responses, increases in BDNF and changes in the ventral HPC activity when compared to non-ketamine controls (Ryan et al. 2022). Another rat study found ketamine administered 24 h prior to FS treatment blocked stress-induced glutamate release and rescued dendritic retraction in the prelimbic PFC and re-stabilised glutamate dysfunction (Sala et al. 2022).

Ketamine’s impact on synaptic plasticity through NMDA receptor inhibition and the subsequent activation of downstream signalling pathways is well-established, although recent research has found that it also leads to changes in calcium-dependent transcription factors such as NFATc4. A recent rodent study included within this review applied prophylactic chronic administration of R-ketamine to determine its inhibitory effect on inflammation and incorporated lipopolysaccharide treatment, a commonly used model for systemic inflammation. It found that the nuclear factor of activated T-cells 4 (NFATc4) signalling pathway contributed to the prophylactic effects of R-ketamine in the PFC, reducing depression-like behaviour and systemic inflammation when applying the forced swim test (FST) (Ma et al. 2022). NFATc4 is a member of the NFAT family of transcription factors crucial in the regulation of gene expression in synaptic plasticity. It is also associated with learning and memory and the regulation of the expression of inflammatory cytokines.

These findings highlight ketamine’s potential as a prophylactic PTSD treatment, showing its ability to reduce fear responses, block stress-induced glutamate release, and preserve dendritic structure in rodent studies. Initial findings have identified NFATc4 activation as a key transcription factor to its neuroprotective and anti-inflammatory effects. However, its clinical use as a preventive measure requires careful evaluation, as the risks may outweigh its current use as a post-trauma intervention.

### Immediate pharmacodynamic effect of ketamine

**Fig. 4:**
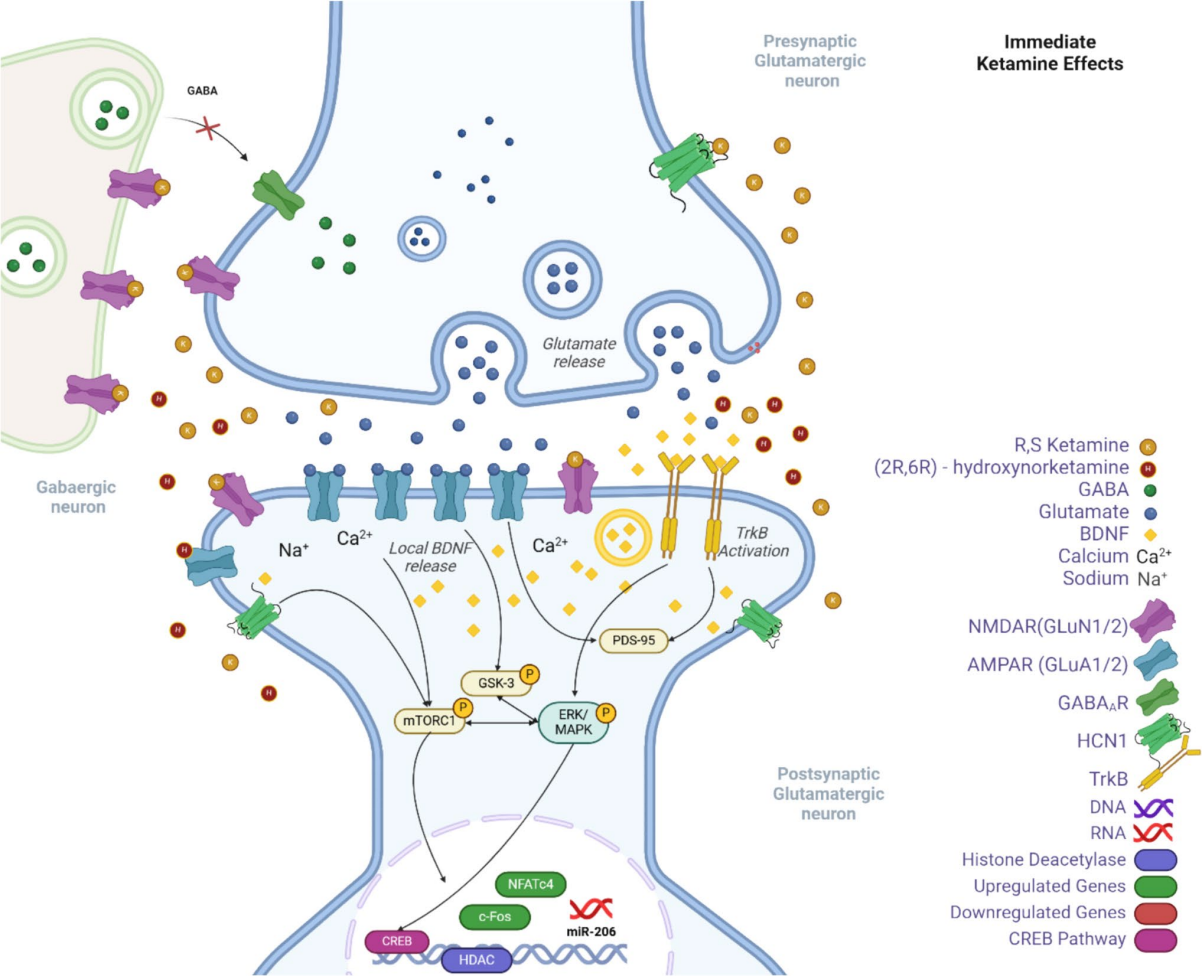
Immediate molecular effects of ketamine. Findings from this review indicate that ketamine inhibits NMDA receptors on GABAergic interneurons, reducing GABA release and leading to the disinhibition of presynaptic glutamatergic neurons, which increases glutamate release. The elevated glutamate activates AMPA receptors on the post-synaptic membrane, facilitating the influx of sodium (Na+) and calcium (Ca2+) ions, essential for downstream signaling. The rise in intracellular calcium promotes the release of BDNF, which binds to TrkB receptors, activating pathways such as mTORC1 and ERK/MAPK. These pathways are critical for synaptic plasticity, enhancing protein synthesis, and promoting synaptic growth and gene transcription. The inhibition of GSK-3 and HDACs further supports these processes by modulating neuroplasticity and gene expression. Additionally, PSD-95 is depicted as stabilising and localising NMDA and AMPA receptors at the postsynaptic density, ensuring effective synaptic transmission. Hyperpolarisation-activated cyclic nucleotide-gated channel 1 (HCN1) activity is down-regulated, affecting neuronal excitability in response to BDNF signaling. Transcription factors such as CREB (cAMP response element-binding protein) and c-Fos, along with miR-206, regulate the expression of genes involved in synaptic plasticity and stress responses, contributing to ketamine’s rapid antidepressant effects. Created in BioRender. Biodiscovery, C. (2024) https://BioRender.com/j72m847

Ketamine’s immediate effects have been primarily attributed to its initial inhibition of NMDA receptors at the phencyclidine site within the ionotropic channel on GABAergic interneurons (formed by the interaction of GluN1 and GluN2 subunits), leading to a net increase in glutamatergic transmission (Milton et al. 2013). This review identified several mouse studies that showed increases in GABA, glutamate and glutamine levels supporting this existing tenet (Palucha-Poniewiera 2018; Schobel et al. 2013; Weckmann et al. 2019). Although studies within this review also identified that NMDA receptor metabolite modulators putrescine and serine were also elevated within 2 h of administration within the HPC (Forti et al. 2023; Weckmann et al. 2019). By blocking NMDA receptors on GABAergic interneurons, ketamine reduces GABA release, leading to disinhibition of excitatory neurons and further enhancing glutamate release. Increases in glutamate activity quickly enhances the function of AMPA receptors, crucial for synaptic potentiation and effective neural communication. These same mouse studies have also identified increased AMPA receptor activation and GluA2 subunit protein levels (Weckmann et al. 2019). GluA2 is critical in determining the calcium permeability of AMPA receptors. AMPA receptors, including those with GluA2, are central to synaptic plasticity mechanisms, such as long-term potentiation (LTP) and long-term depression (LTD), crucial for learning and memory (Cull-Candy et al. 2006) (Fig. 4).

A recent rodent study also examined GluA1 AMPA subunit interaction with BDNF and the PSD-95 protein expression pathway by the intracerebroventricular administration of HNK to determine its effect on the PFC (Li et al. 2022). The GluA1-BDNF signalling pathway involves synaptic activity-induced phosphorylation of the GluA1 subunit of AMPA receptors, enhancing their trafficking to the synaptic membrane and strengthening synaptic connections. Concurrently, BDNF released during synaptic activity binds to TrkB receptors, activating downstream signalling pathways that promote synaptic plasticity and the transcription of genes involved in synaptic growth and maintenance (Yaprak et al. 2024). PSD-95 is also integral in anchoring and organising synaptic receptors, such as NMDA and AMPA receptors, at the postsynaptic density of excitatory synapses in the brain. When BDNF activates TrkB receptors, it can enhance the expression and function of these receptors at the synapse. PSD-95 helps to stabilise these receptors and ensure their proper localisation at the postsynaptic site (Sheng 2001). Additional rodent models have supported these findings with the detection of increased levels of BDNF from the acute administration of ketamine through IP or IV applications (Woelfer et al. 2020).

Mouse studies have investigated region-specific BDNF increases during the acute stage of ketamine therapy, showing increased BDNF expression in the mPFC and HPC and hypo-methylation of promoter region exon IV (Ju et al. 2017). These findings were replicated in another study identifying increases in BDNF mRNA within 30 min of IV ketamine administration (peaking at 6 h post-administration), with significant increases in exon IV detected during therapy (Choi et al. 2017). Additional studies have supported changes in BDNF levels during acute ketamine administration in the mPFC and HPC (Zhang et al. 2015).

Correlations have been investigated through further rodent models measuring changes in BDNF and hyperpolarisation-activated cyclic nucleotide-gated channel 1 (HCN1) in the PFC following acute ketamine administration. The HCN channel plays a significant role in the regulation of neuronal excitability and synaptic transmission (Zhou et al. 2013). BDNF signalling has been shown to influence the gating properties and expression levels of HCN channels in neurons (Ni et al. 2020). In PTSD, where BDNF levels may be reduced, this can lead to altered HCN1 activity, which may affect neuronal excitability and synaptic plasticity (Hou et al. 2018; Kim and Johnston 2018). This study found that whilst BDNF increased, HCN1 decreased in the PFC (Hou et al. 2018).

c-Fos is another early gene marker commonly used to identify neuronal activation through the extracellular signal-regulated kinase / mitogen-activated protein kinase (ERK/MAPK) signalling pathway which is involved in regulating various cellular processes, including proliferation, differentiation, and synaptic plasticity alongside increases in BDNF (Zhang et al. 2019). A recent study found increases in the c-Fos transcription factor in the basolateral amygdala (BLA) via HNK intracerebroventricular infusion resulting in the re-regulation of fear memory in mice (Xu et al. 2023).

Other modifiers effected by acute ketamine administration include decreases in glycogen synthase kinase-3 (GSK-3) and histone deacetylase (HDAC) immunoreactivities within 24 h of ketamine administration in the dorsal striatum (DS), dentate gyrus (DG), CA1 and CA3 region of the HPC and the PFC while there was a concurrent increase in BDNF in PFC, DG, CA1, and CA3 areas (Viana et al. 2020). HDAC’s are enzymes that remove acetyl groups from histones, leading to chromatin condensation and reduced gene transcription. HDACs are epigenetic regulators that influence synaptic plasticity, memory formation, and stress responses. They also play roles in neurodevelopment and neuroprotection (Matsumoto et al. 2013). GSK-3 is a critical serine/threonine kinase that regulates glycogen synthesis, cell signalling, and neuroplasticity (Jaworski et al. 2019). In PTSD, GSK-3’s dysregulation affects synaptic plasticity, neurogenesis, and stress response pathways, leading to maladaptive memory consolidation and heightened stress reactivity, making it a potential target for therapeutic intervention (Liu et al. 2013).

Ketamine administration in rodent models has also identified a reduction of GSK-3β levels in the hypothalamus, a brain region crucial for regulating stress responses and mood. By inhibiting GSK-3β, ketamine may enhance neuroplasticity, improving synaptic connectivity, and remodulating the transcription of genes involved in cell growth and survival (Hu et al. 2023).

Additional studies measuring alterations in the HPA found changes in corticotropin-releasing hormone (CRH) levels within 24 h of ketamine infusion (Radford et al. 2018). The HPA regulates the body’s response to stress by secreting CRH from the hypothalamus, which stimulates the release of adrenocorticotropic hormone (ACTH) from the anterior pituitary, in turn acting on the adrenal glands to secrete glucocorticoids into the blood stream (Tafet and Nemeroff 2020). PTSD sufferers have been shown to have lower CRH levels, potentially due to an enhanced HPA negative feedback loop (Ramos-Cejudo et al. 2021). One study detected elevated progesterone levels as a precursor to corticosterone, indicating ketamine was stimulating the HPA and suggesting a release of corticosterone from the adrenal gland (Radford et al. 2020).

Immune markers are also widely investigated mechanisms of influence for ketamine therapy. Whereby rodent models have identified decreases in interleukin (IL)−6 and IL-1β levels in the HPC as a consequence of acute IP ketamine administration (Valenza et al. 2024). IL-6 and IL-1β are pro-inflammatory cytokines that play significant roles in the brain’s immune response in PTSD (Lindqvist et al. 2014; Rudzki 2022). Additional research also discovered varied expression of tumour necrosis factor-alpha (TNF-α) a key mediator of neuroinflammation in the HPC, based on ketamine dose and duration (Li et al. 2017; Yang et al. 2022).

Post-transcriptional effects have also been detected within the first 24 h of ketamine administration, with one study identifying the down-regulation of miR-206 (a critical BDNF inhibitor) in the HPC within 12 h of ketamine treatment (Yang et al. 2014). Although significant, very little research has focussed on the acute effects of ketamine on post-transcriptional pathways.

### Sustained pharmacodynamic effect of ketamine

Ketamine’s influence on calcium signalling pathways plays a critical role in both intercellular and intracellular processes, leading to long-lasting changes in gene expression. By elevating intracellular calcium levels, ketamine initiates signalling cascades which result in the phosphorylation of key transcription factors and epigenetic regulators (Kawatake-Kuno et al. 2021). This activity-dependent modulation of gene expression is a critical mechanism through which ketamine induces rapid changes in neuronal function and synaptic connectivity, as well as longer-term molecular changes in synaptic plasticity and functional connectivity, contributing to its long-term effects on PTSD (Green and Cote 2009; Lisek et al. 2020).

In the weeks following treatment, many of the ketamine studies continued to detect molecular responses to the therapy (Choi et al. 2017; Donahue et al. 2014; Duclot et al. 2016; Girgenti et al. 2017; Gou et al. 2023; Hu et al. 2023; Ito et al. 2015; Ju et al. 2017; Lee et al. 2019; Li et al. 2017; Ma et al. 2022a; Ma, Zhang, Ma et al. 2022a, b; Ryan et al. 2022; Sala et al. 2022; Sun et al. 2022; Viana et al. 2020; Weckmann et al. 2019; Woelfer et al. 2020; Yang et al. 2014, 2022; Zhang et al. 2015). An underlying molecular marker for PTSD is the dysregulation of GABA and glutamate in brain regions responsible for emotional regulation and memory. One mouse study measuring GABA and glutamate in the short- and long-term post-ketamine administration found significant increases in AMPAR subunit GluA2 activity in the HPC (even after 72 h) resulting in decreased GABAergic inhibitory neurotransmission and leading to increased excitatory neuronal activity (Weckmann et al. 2019).

Activation of the mammalian target of rapamycin (mTOR) pathway has been found to be a crucial component of ketamine’s rapid antidepressant effects (Xu et al. 2021). Upon ketamine administration, the increased extracellular glutamate levels activate AMPA receptors, stimulating downstream signalling pathways, including mTOR. The activation of mTOR leads to a series of intracellular events that culminate in enhanced synaptic protein synthesis and synaptogenesis. A benchmark mouse study combined the AFC model with post intervention training techniques to examine several molecular targets and specific pathways for ketamine’s effect on PTSD. The first experiment combined ketamine and extinction exposure learning and retrieval to detect increased mTORC1 levels in the mPFC (Girgenti et al. 2017). This was followed by the infusion of the selective mTORC1 inhibitor rapamycin into the mPFC, which blocked ketamine’s effects on extinction. Next, ketamine combined with extinction training was found to increase c-Fos in the mPFC. Finally, the administration of a glutamate-AMPA receptor antagonist (NBQX) blocked ketamine’s effects (Girgenti et al. 2017).

Another rodent study tested HNK on SPS&FS rodents through ICBV injection into the NAc. Here, when the changes in BDNF, mTOR, and PSD-95 were measured, it was identified that the SPS&FS group exhibited PTSD symptoms with reduced levels of these proteins and damaged synaptic morphology (Gou et al. 2023). The administration of 50µM (2R,6R)-HNK restored protein levels and synaptic ultrastructure in the NAc, improved PTSD symptomology (including locomotor behaviour and social interaction) and reregulated BDNF/mTOR-mediated synaptic structural plasticity in the Nac. This study supports the mechanism of action that ketamine-induced mTOR activation enhances the synthesis of proteins necessary for maintaining long-term synaptic changes (Gou et al. 2023).

A study of fear generalised mice showed a lower level of BDNF and a higher level of GluN2B protein in the BLA and infralimbic PFC, and this was reversed by a single administration of ketamine (Asim et al. 2020). Ketamine, over 22 h post-conditioning, reduced fear generalisation significantly, with this effect lasting 2 weeks. Ketamine was also found to increase BDNF and decrease GluN2B (Asim et al. 2020). Another rodent study revealed increases in BDNF and PSD-95 in the AMY and HPC reversing cognitive impairment and reducing fear memory well past ketamine’s elimination from the host body (Sun et al. 2022). Additional rodent studies have also supported the sustained renormalisation of BDNF, including altered BDNF mRNA levels measured up to three months post-ketamine therapy (Woelfer et al. 2020; Zhang et al. 2015).

In a study of the GSK-3β signalling pathway, ketamine was found to reduce the protein levels of GSK-3β, GR, p-GSK-3β (phosphorylated form of GSK-3β) and alter the ratio of p-GSK-3β to GSK-3β (Hu et al. 2023). Correspondingly, gene expression of GSK-3β, glucocorticoid receptor (GR), BDNF, and FkBP5 decreased in the SPS-Ket group compared to the SPS control group. Ketamine ameliorated PTSD symptoms by reducing protein and gene expression levels of the GSK-3β pathway, thereby improving anxiety-like behaviour in SPS-exposed rats (Hu et al. 2023).

Novelty seeking behaviour in rats was also found to predict fear response and extinction differences. Exposure to ketamine was observed to disrupt contextual fear reconsolidation, reducing fear memory well past the time of drug excretion (Duclot et al. 2016). Ketamine was also found to down-regulate early growth response 1 (Egr1) in the HPC CA1 area of mice. Erg1 encodes a zinc-finger transcription factor that binds to specific DNA sequences to regulate the expression of target genes involved in neuronal plasticity, learning, and memory. The same study found that ketamine also upregulates BDNF mRNA in the prelimbic and infralimbic cortices (Goldberg et al. 2011).

Another mouse model applying foot shock treatment (FS) and utilising oral ketamine (compared to imipramine, a control antidepressant) found significant increases in glial fibrillary acidic protein (GFAP) and BDNF expression 30 days post-ketamine administration compared to impramine (Viana et al. 2020). GFAP is a filament protein that is primarily expressed in astrocytes. An increase in GFAP expression in the PFC and striatum suggests that ketamine may counteract the loss of astrocytes observed in depression. Further, these changes, alongside a decrease in HDAC and GSK-3 immunoreactivities, were also identified in the PFC, DG, CA1 brain regions, leading to long-lasting antidepressant-like effects in ketamine-treated mice compared to the imipramine-treated mice (Viana et al. 2020).

A mouse study applying the AFC model measured the development of silent synapses. Silent synapses are synaptic connections that are initially inactive due to the lack of functional AMPA receptors but have the potential to become active through synaptic plasticity mechanisms, playing a crucial role in brain development and the adaptive changes associated with learning and memory (Ito et al. 2015). They found that observational fear exposure altered synaptic transmission in the dmPFC generating emotional ruminating and increasing silent synapses in the PFC-AMY pathway (Ito et al. 2015). When administered, ketamine abolished silent synapse formation in the prefrontal-AMY pathway and prevented silent synapse formation, hence preventing the enhancement of fear learning (Ito et al. 2015).

The ERK phosphorylation pathway is also essential for synaptic plasticity and LTP processes underlying memory formation and storage (Wen et al. 2017). A rodent study utilising the SPS&FS PTSD formation model found that the dysregulation of the ERK pathway can lead to maladaptive memory consolidation, contributing to persistent traumatic memories characteristic of PTSD. In this study, they measured a decrease in ERK phosphorylation in the AMY and PFC, alongside GluA1 and PSD-95 decreases, leading to glutamate abnormalities in these brain regions. They compared fluoxetine (an SSRI antidepressant) with ketamine to determine their therapeutic effectiveness and found that ketamine normalised the glutamate and ERK phosphorylation-related pathway (including the PSD protein abnormalities in the AMY and PFC) after 24 h (Lee et al. 2019). This, effectively reversed the stress-induced changes in the synaptic proteins bought on by the model, as opposed to the minimally observed stress change in the fluoxetine-treated mice (Lee et al. 2019).

Moreover, ketamine’s sustained influence extends to the modulation of the inflammatory response, a component increasingly recognised as crucial in the pathogenesis of PTSD (Yaprak et al. 2024; Zhan et al. 2020). By attenuating pro-inflammatory cytokines and activating anti-inflammatory pathways, ketamine can mitigate the inflammatory burden on the brain, which is often exacerbated by chronic stress and trauma. This anti-inflammatory action may contribute to the sustained therapeutic effects by preventing further neuronal damage and promoting recovery processes in neural tissues that are vital for cognitive and emotional functions (Tan et al. 2017).

A study administering S-ketamine to SPS-exposed rodents and followed up to 12 days post-infusion, found that SPS-exposed rats exhibited TNF-α, IL-1β, p-NFkB, and NF-kB upregulation in the dorsal striatum and periaqueductal grey (PAG), rather than ACC and PFC. S-Ketamine treatment reduced microglia activation and reversed TNF-α, IL-1β, p-NFkB and NF-kB pro-inflammatory cytokines in the dorsal striatum (Yang et al. 2022). This contributed to the reduction of PTSD symptoms, including heightened stress responses.

The only long-term stress induced mouse model administered a daily chronic dose of 60 mg/kg ketamine over a 6-month period and found reduced mRNA levels of IL-6 and IL-1β and TNF-α in the HPC. The study provides new insights into how prolonged chronic doses of ketamine may contribute to changes in the levels of IL-6, IL-1β, and TNF-α, and how this might contribute to its neurotoxic effects. These findings highlight the potential role of inflammatory processes in the adverse effects of ketamine on the brain. Additionally, ketamine was found to induce spatial memory deficit and reduced anxiety (Li et al. 2017).

### Ketamine’s sustained genetic and epigenetic influence

Ketamine’s sustained effects on PTSD mediated via epigenetic and genetic pathways represent a critical area of research that may explain the sustained therapeutic benefits observed in patients. Ketamine has been shown to decrease DNA methylation at specific promoter regions, leading to increased expression of genes that support neuronal growth and synaptic plasticity (Rump and Adamzik 2022). These epigenetic changes provide a lasting impact on neuronal circuits, which is crucial for the sustained antidepressant effects observed with ketamine treatment.

One of the primary targets of ketamine’s effect on DNA methylation is the BDNF gene. Ketamine administration has been shown to decrease methylation at the promoter region of the BDNF gene, leading to increased expression of BDNF (Ribeiro-Davis et al. 2024). This epigenetic modification is important because BDNF is vital for synaptic plasticity, neuronal survival, and cognitive function (Voisey et al. 2019; You and Lu 2023). Furthermore, a mouse study examining the mechanism of action for DNA methyltransferases (DNMTs) on BDNF methylation found that inescapable foot shock (IFS) significantly increased levels of DNMT3a and DNMT3b. Long-term ketamine treatment (22 days) normalised DNMT3a and DMNT3b levels and also BDNF exon IV gene methylation, leading to increased BDNF expression in mPFC and HPC, alleviating fear relapse (Ju et al. 2017).

Another recent study administering R-ketamine in chronic social defeat stress (CSDS) susceptible mice activated BDNF and inhibited methyl-CpG-binding protein 2 (MeCP2) expression in microglia. MeCP2 is an epigenetic regulator that binds to methylated DNA and recruits HDACs, leading to transcriptional repression. Calcium signalling can phosphorylate MeCP2 and reduce its binding affinity for methylated DNA, thereby altering gene expression. Ketamine’s effect on calcium signalling can modulate the activity of MeCP2, leading to changes in the expression of genes involved in synaptic plasticity and neuronal function (Buchthal et al. 2012). The suppression of MeCP2 may be a key mechanism through which R-ketamine exerts longer-term antidepressant effects in the PFC (Ma et al. 2022). Additionally, and within this same study, microRNA-132-5p (miR-132-5p) has been identified as a regulator of both BDNF and MeCP2 expression in the PFC. Results indicated that miR-132-5p attenuated altered expression due to ketamine administration, restoring levels of BDNF, MeCP2 and TGF-b1 in the PFC, and improving depression like behaviours (Ma et al. 2022).

Ketamine significantly influences histone acetylation, particularly by inhibiting HDACs, especially HDAC5 (Choi et al. 2015). HDAC5, a critical enzyme involved in the regulation of gene expression through chromatin remodelling, has recently been implicated in the sustained modulation of BDNF expression, particularly in the HPC (a brain region essential for learning and memory). A rodent study demonstrated that the knockdown of HDAC5 using lentiviral vectors in the DG of the rat HPC resulted in a significant reduction in HDAC5 mRNA levels. This knockdown led to an increase in BDNF mRNA and protein levels, suggesting that HDAC5 negatively regulates BDNF expression. Furthermore, the administration of ketamine was shown to enhance BDNF expression in the DG, an effect that was blocked by HDAC5 knockdown indicating that HDAC5 is a crucial mediator of ketamine’s action on BDNF expression (Choi et al. 2017).

Research has also shown that epigenetic mechanisms, including histone modifications like H3K27me3 (a trimethylation of the 27th lysine residue on histone H3), play a significant role in the pathophysiology of PTSD (Ell et al. 2024). For example, ketamine administration has been shown to increase BDNF expression in the rat brain, potentially through alterations in H3K27me3 (Duclot et al. 2016). Furthermore, miRNAs which are also regulated by epigenetic mechanisms, have been implicated in the antidepressant effects of ketamine. Previously reported, miR-132-5p is upregulated in PTSD participants and is influenced by histone modifications (like H3K27me3), thereby affecting PTSD symptomology and treatment response (Nie et al. 2021). A rodent model observed the disruption in reconsolidation of fear memories after ketamine treatment, alongside changes in histone modifications like H3K27me3, providing strong evidence as to how histone modifications lead to therapeutic effects in PTSD (Girgenti et al. 2017).

**Fig. 5:**
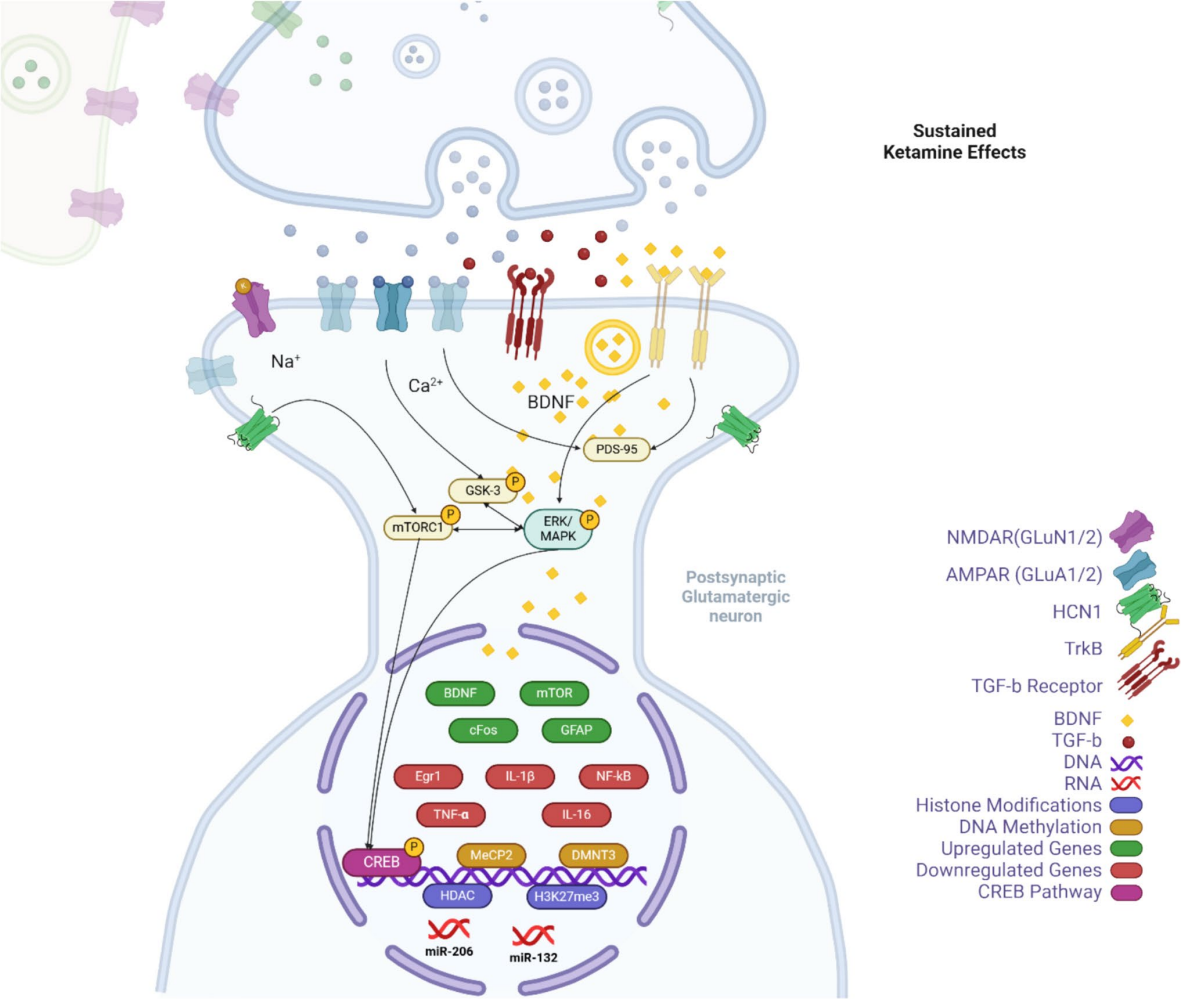
Sustained molecular effects of ketamine. Findings from this review indicated that ketamine primarily acts as an NMDAR (GluN1/2) antagonist, reducing calcium influx and modulating AMPAR (GluA1/2) activity. It also influences the long-term up-regulation of BDNF, which binds to TrkB receptors, activating downstream signalling cascades such as the ERK/MAPK and mTORC1 pathways. Additionally, GSK-3 is inhibited, further supporting the enhancement of synaptic plasticity and contributing to ketamine’s antidepressant effects. The CREB pathway, activated by ERK/MAPK and mTOR signalling, promotes the transcription of genes essential for synaptic function. MeCP2, along with histone modifiers (HDACs, H2K27me3) and DNA methyltransferases (DNMT3), modulates chromatin structure, leading to long-term gene expression changes through histone modifications and DNA methylation. Ketamine also influences the long-term expression of specific microRNAs, such as miR-206 and miR-132, resulting in the up-regulation of genes that favour neuroplasticity and neurogenesis. Ketamine also effects the long-term down-regulation of pro-inflammatory pathways, including IL-1β, TNF-α, and NF-κB, which are typically up-regulated in stress and depression, thus contributing to its anti-inflammatory and antidepressant effects. TGF-β receptor signalling may also contribute to the overall neuroprotective effects of ketamine. Figure generated using Biorender.com

While ketamine has been shown to affect miRNAs, much of that research has focussed primarily on the rapid and sustained antidepressant effects of ketamine (placing it beyond the scope of this review) (Girgenti et al. 2017). Ketamine administration has been implicated in the attenuation of miR-132-5p, which raised reduced levels of BDNF and TGF-b1 in the PFC and reduced PTSD symptoms (Ma et al. 2022). Another study identified the down-regulation of miR-206 in the rat HPC, not only during the ketamine treatment, but well after 24 h of expulsion. miR-206 is considered a critical BDNF inhibitor in the HPC (Yang et al. 2014). Ketamine has also been linked to attenuated apoptosis induced by overexpression of miR-206 in the HPC associated with PTSD symptoms. High miR-206 expression increased calcium currents and transient potassium currents in hippocampal neurons (Guan et al. 2021) (Fig. 5).

### Neuromodulation

This review identified research focussing on postsynaptic currents (PSCs). PSCs are also essential for understanding the intricacies of synaptic transmission and plasticity, especially when exploring the pathophysiology of PTSD and the therapeutic mechanisms of ketamine. In PTSD, the brain’s stress response system is often dysregulated, leading to alterations in neurotransmitter release and receptor sensitivity (Somvanshi et al. 2018). This dysregulation can manifest as changes in PSCs, particularly in brain regions associated with fear, memory, and emotional regulation, such as the AMY, HPC, and PFC (Kim et al. 2020). Miniature excitatory postsynaptic currents (mEPSCs) and spontaneous excitatory postsynaptic currents (sEPSCs) are two types of PSCs that provide insights into synaptic function (Yang et al. 2018). A study examining the effects of forced swim stress on adult rats found significant neuro-architectural changes, such as the retraction of pyramidal neuron apical dendrites in PFC layers II-III, which was prevented by chronic pretreatment with desipramine. Furthermore, ketamine modulated glutamatergic transmission, stabilising glutamate dysfunction induced by this acute stress model. Ketamine blocked the stress-induced glutamate release, restoring glutamate release to control levels in hypofunctional synapses and dampened glutamate efflux in hyperfunctional synapses in the prelimbic prefrontal cortex (PL-PFC), stabilising glutamate dysfunction within 24 h. Within the same study, mEPSCs were recorded in pyramidal neurons in the PL-PFC, reversing dendritic atrophy and spine loss resulting in fear extinction behaviours (Sala et al. 2022). These findings underscore the importance of PSCs as synaptic mechanisms underlying stress responses and the therapeutic effects of ketamine.

### Comprehensive approach to PTSD treatment with ketamine

Ketamine has been found to aid in the reduction of fear memories, a crucial aspect of PTSD treatment, by altering synaptic signalling pathways that are vital for the consolidation and retrieval of fear memories (Milad et al. 2009). By blocking NMDA receptors, ketamine enhances the activity of AMPA receptors, which in turn promotes synaptic plasticity in key regions of the brain, such as the PFC, HPC, and AMY. This process leads to the increased expression of genes related to neuroplasticity, including BDNF, PSD-95, and mTOR, supporting the restructuring of synaptic connections necessary for the effective extinction of fear (Duclot et al. 2016).

Medication-assisted PTSD therapy utilising ketamine, however, requires further investigation into the disrupted processes involved in memory extinction. Expanding preclinical research to explore the role of NMDA receptors in the modulation, acquisition, and storage of memories before and after consolidation will help determine whether ketamine’s targeting of these receptors can effectively treat PTSD. For example, ketamine treatment during stress exposure has been shown to prevent the loss of dendritic spine density in the CA3 region and DG of the HPC, while also increasing markers of synaptic plasticity, such as synapsin 1, PSD-95, and mGluR1 in stressed rats (Garfinkel et al. 2014).

In addition to its effects on synaptic function, ketamine may also trigger processes that contribute to the reconsolidation of memories, thereby promoting neurogenesis and the formation of new synapses. Research has demonstrated that ketamine infusion after fear learning can reduce behaviours associated with fear in mice and reverse reductions in HPC BDNF levels caused by chronic stress (Asim et al. 2020). Moreover, a single infusion of ketamine has been shown to alleviate PTSD-like symptoms in rats by elevating BDNF levels in the PFC (Lee et al. 2019). These findings suggest that integrating ketamine with psychotherapy at critical time points during memory reconsolidation could enhance the extinction of fear and reduce flashbacks. Additionally, developing safe protocols for the chronic, low-dose use of ketamine could improve its effectiveness in treating PTSD.

Ketamine, a promising agent for PTSD treatment, has been studied in combination with various psychotherapies to extend and amplify its therapeutic effects (Philipp-Muller et al. 2023). Both rodent and human studies have indicated that ketamine can influence fear extinction and memory reconsolidation processes crucial for mitigating the impact of traumatic memories, a core aspect of prolonged exposure (PE) therapy (commonly used to treat PTSD) (Duek et al. 2019). Preclinical studies suggest ketamine can enhance the recall of extinction learning and reduce the likelihood of fear renewal, potentially increasing the effectiveness of PE (Difede et al. 2022; Hammoud et al. 2020; Shiroma et al. 2024). By enhancing learning, ketamine may help patients process and integrate therapeutic experiences during PE therapy sessions more effectively, leading to improved treatment outcomes for PTSD.

Initial small-scale studies have shown positive results when ketamine is used to augment PE therapy, indicating that it could be a valuable addition to traditional PTSD treatments, especially for individuals who do not respond well to standard therapies. In clinical settings, ketamine has been explored as an adjunct to PE therapy in veterans with PTSD, with ongoing research aimed at determining the efficacy and safety of this combined treatment approach (Shiroma et al. 2024).

Ketamine has also been examined for its potential to enhance the effects of extinction therapy, another core element of PTSD treatment (Glavonic et al. 2024). One study found that ketamine could reduce PTSD symptoms by targeting traumatic memories encoded in specific brain regions, such as the BLA. This effect was observed when ketamine was administered during a critical re-exposure window, reducing the activity of BLA engram cells associated with traumatic memories (Li et al. 2024).

Another therapeutic approach involves combining ketamine with mindfulness-based cognitive therapy, specifically Trauma Interventions using Mindfulness-Based Extinction and Reconsolidation (TIMBER). This combination has significantly extended the duration of therapeutic response, with studies reporting sustained effects for up to 34 days (compared to 16 days with placebo) (Pradhan et al. 2018).

Combining ketamine with psychotherapy not only prolongs its therapeutic effects, but also addresses the issue of high treatment-resistance seen in PTSD patients, where 35–50% of individuals do not respond to standard treatments (Weber et al. 2021). The synergistic effect of ketamine and psychotherapy is thought to involve modulation of the glutamate system and enhancement of synaptic plasticity, both of which are essential for altering trauma-related memories and fostering new learning. Despite these promising results, the evidence remains preliminary and further research is needed to establish the most effective protocols and fully understand the underlying molecular mechanisms. Overall, while ketamine alone offers rapid and sustained symptom relief, its combination with psychotherapy appears to provide a more robust and enduring therapeutic benefit for PTSD patients, underscoring the potential of this integrated treatment approach.

### Limitations

The review highlights several key limitations in the current research of ketamine’s effects on PTSD. A major limitation is the reliance on animal models, which may not fully translate to human outcomes, particularly regarding long-term effects. Mice also have a relatively simpler brain structure, which might limit the extrapolation of findings to more complex human brain functions. The heterogeneity of study designs, including variations in dosage, treatment duration, and preclinical versus clinical approaches, further complicates the synthesis of findings and may obscure important differences in ketamine’s therapeutic mechanisms. Furthermore, current research tends to emphasise ketamine’s positive effects, with limited attention given to potential adverse outcomes, such as dissociation, addiction, or cardiovascular risks. These factors are critical when evaluating ketamine’s clinical application for PTSD treatment.

Moreover, while the review distinguishes between ketamine’s immediate and sustained effects, there was only one rodent study with a focus on the longer-term effects of chronic daily use of ketamine (> 6 months) publishing adverse effects. There is a distinct lack of in-depth exploration into rodent and pre-clinical trials essential for understanding the longer-term implications of repeated ketamine administration and its efficacy, tolerances or maintenance schedules where thresholds between efficacy and adverse effects are observed.

The emerging potential of ketamine as a prophylactic treatment or in combination with psychotherapy is promising but remains underexplored, especially in human studies. Additionally, more research is needed to clarify how ketamine’s effects on epigenetic and inflammatory markers may vary among individuals, which is essential for developing personalised treatment strategies. These limitations suggest that future research should focus on addressing these gaps to better understand ketamine’s full therapeutic potential and safety in treating PTSD.

## Conclusion

The reviewed evidence outlines the molecular mechanisms that drive ketamine’s immediate effects (while it remains active in the body) as well as the mechanisms that trigger sustained effects (through intra- and inter-cellular pathways crucial for sustained gene expression changes). Changes in key brain regions (HPC, AMY, PFC and BLA) have been found to reduce the phenomenology of PTSD symptoms, including fear extinction and memory reconsolidation, reducing depression and anxiety-like behaviours and systemic inflammation.

The immediate pharmacodynamics of ketamine reveal its profound and complex impact on neural and molecular processes, particularly in the context of treating PTSD. Although the inhibition of NMDA receptors and consequential excitation of AMPA receptor activity leads to rapid glutamate release and synaptic potentiation, there are many additional key mechanisms underlying ketamine’s effects on PTSD phenomenology. The influence on NMDA and AMPA receptor subunits, GLuN1/2 and GLuA1/2, however, remains key to synaptic plasticity. Also, the impact of acute ketamine administration on BDNF mRNA and its subsequent protein (and the downstream effects on relevant signalling pathways) is formidable. Further influence of ketamine’s modulation of critical pathways includes GSK-3, PSD-95 and HCN channels. Its influence also includes key transcription factors (like c-Fos) and epigenetic regulators (like HDAC), alongside post-transcriptional modifiers (like miR-206), that can contribute to improved neuroplasticity and synaptic connectivity. The observed changes in inflammatory markers and epigenetic regulation underscore ketamine’s short-term therapeutic multifaceted influence on brain function, emphasising ketamine as a rapid and effective treatment for PTSD.

Ketamine’s sustained therapeutic effects on PTSD follow on from these initial effects and are deeply rooted in its ability to modulate many of the same key molecular pathways at an epigenetic and transcriptional level and through synaptic processes. Ketamine influences calcium signalling, a critical pathway that leads to the phosphorylation of transcription factors and epigenetic regulators, resulting in sustained changes in gene expression. This modulation enhances the expression of key synaptic proteins such as AMPA receptor subunits (e.g., GLuA2), BDNF, and postsynaptic density proteins (e.g., PSD-95), all of which are crucial for maintaining synaptic plasticity and functional connectivity. Additionally, ketamine’s activation of the mTOR pathway has been shown to promote synaptogenesis and protein synthesis, reversing synaptic deficits induced by chronic stress. Molecular studies have further demonstrated that ketamine reduces the dysregulation of GABAergic and glutamatergic systems in PTSD, normalising neurotransmission in critical brain regions like the mPFC and HPC, leading to improved behavioural outcomes.

Moreover, ketamine’s impact extends beyond immediate synaptic modulation to include significant epigenetic and inflammatory responses. By decreasing DNA methylation at specific promoter regions, particularly those associated with the BDNF gene, ketamine facilitates sustained gene expression changes that are vital for long-term synaptic stability. Additionally, ketamine has been shown to influence histone modifications (including the inhibition of HDAC, MeCP2 and H3K27me3) alongside post-transcription regulators (like miR-132 and miR-206), which further supports the enhancement of synaptic plasticity and cognitive function. Ketamine’s anti-inflammatory actions, evidenced by the attenuation of pro-inflammatory cytokines such as TNF-α and IL-1β, also play a crucial role in mitigating the chronic inflammatory state often associated with PTSD. The reduction in microglial activation and normalisation of neuroinflammatory markers contribute to the protection and recovery of neural circuits involved in emotional regulation and memory.

Further novel approaches, although based on animal models and still in their infancy, identify ketamine as a potential prophylactic treatment prior to trauma. These have yielded positive results through buffering neurotransmitter modulations in GABAergic, BDNF and immune responses in trauma-based exposure models.

Integrating ketamine with psychotherapy during memory reconsolidation presents a promising strategy for PTSD treatment. Ketamine’s unique multifaceted molecular effects underscore its potential as a long-term robust therapeutic agent for PTSD and offer sustained relief through its comprehensive impact on both synaptic and genetic-epigenetic mechanisms. By targeting both the biological and psychological aspects of memory, this approach offers a comprehensive method that could lead to more effective and sustained relief from PTSD symptoms. Future studies should focus on refining these methods, optimising timing, dosage, and the combination of therapeutic techniques to maximise patient outcomes. Understanding these molecular mechanisms not only offers valuable insights into ketamine’s therapeutic effects but also guides the development of new, more patient-centred treatment strategies for PTSD, potentially shaping future research, clinical practice, and policy.

## Supplementary Information

Below is the link to the electronic supplementary material.

Supplementary file 1 (XLSX 14.7 KB)

## Data Availability

All data analysed in this systematic review were obtained from previously published studies, which are publicly available. A full list of included studies and their sources is provided in the manuscript and supplementary material.
